# Probing the Genome-Scale Metabolic Landscape of Bordetella pertussis, the Causative Agent of Whooping Cough

**DOI:** 10.1128/AEM.01528-17

**Published:** 2017-10-17

**Authors:** Filipe Branco dos Santos, Brett G. Olivier, Joost Boele, Vincent Smessaert, Philippe De Rop, Petra Krumpochova, Gunnar W. Klau, Martin Giera, Philippe Dehottay, Bas Teusink, Philippe Goffin

**Affiliations:** a Systems Bioinformatics/AIMMS, Vrije University Amsterdam, Amsterdam, The Netherlands; b Molecular Microbial Physiology, Swammerdam Institute for Life Sciences, University of Amsterdam, Amsterdam, The Netherlands; c Centrum Wiskunde & Informatica (CWI), Amsterdam, The Netherlands; d GSK Vaccines, Rixensart, Belgium; e Algorithmic Bioinformatics, Heinrich Heine University, Düsseldorf, Germany; f Center for Proteomics and Metabolomics, Leiden University Medical Center, Leiden, The Netherlands; g Laboratoire de Génétique et Physiologie Bactérienne, IBMM, Faculté des Sciences, Université Libre de Bruxelles (ULB), Gosselies, Belgium; University of Tartu

**Keywords:** Bordetella pertussis, whooping cough, vaccine production, genome-scale metabolic model, rational medium design, constraint-based modeling

## Abstract

Whooping cough is a highly contagious respiratory disease caused by Bordetella pertussis. Despite widespread vaccination, its incidence has been rising alarmingly, and yet, the physiology of B. pertussis remains poorly understood. We combined genome-scale metabolic reconstruction, a novel optimization algorithm, and experimental data to probe the full metabolic potential of this pathogen, using B. pertussis strain Tohama I as a reference. Experimental validation showed that B. pertussis secretes a significant proportion of nitrogen as arginine and purine nucleosides, which may contribute to modulation of the host response. We also found that B. pertussis can be unexpectedly versatile, being able to metabolize many compounds while displaying minimal nutrient requirements. It can grow without cysteine, using inorganic sulfur sources, such as thiosulfate, and it can grow on organic acids, such as citrate or lactate, as sole carbon sources, providing *in vivo* demonstration that its tricarboxylic acid (TCA) cycle is functional. Although the metabolic reconstruction of eight additional strains indicates that the structural genes underlying this metabolic flexibility are widespread, experimental validation suggests a role of strain-specific regulatory mechanisms in shaping metabolic capabilities. Among five alternative strains tested, three strains were shown to grow on substrate combinations requiring a functional TCA cycle, but only one strain could use thiosulfate. Finally, the metabolic model was used to rationally design growth media with >2-fold improvements in pertussis toxin production. This study thus provides novel insights into B. pertussis physiology and highlights the potential, but also the limitations, of models based solely on metabolic gene content.

**IMPORTANCE** The metabolic capabilities of Bordetella pertussis, the causative agent of whooping cough, were investigated from a systems-level perspective. We constructed a comprehensive genome-scale metabolic model for B. pertussis and challenged its predictions experimentally. This systems approach shed light on new potential host-microbe interactions and allowed us to rationally design novel growth media with >2-fold improvements in pertussis toxin production. Most importantly, we also uncovered the potential for metabolic flexibility of B. pertussis (significantly larger range of substrates than previously alleged; novel active pathways allowing growth in minimal, nearly mineral nutrient combinations where only the carbon source must be organic), although our results also highlight the importance of strain-specific regulatory determinants in shaping metabolic capabilities. Deciphering the underlying regulatory mechanisms appears to be crucial for a comprehensive understanding of B. pertussis's lifestyle and the epidemiology of whooping cough. The contribution of metabolic models in this context will require the extension of the genome-scale metabolic model to integrate this regulatory dimension.

## INTRODUCTION

Pertussis, or whooping cough, is a highly contagious respiratory disease caused by the Gram-negative bacterium Bordetella pertussis. Despite widespread vaccination, it remains an important health burden, with an estimated 16 million cases in 2008 (1). As recently as 2013, it was still responsible for 1% of the global mortality (60,000 deaths) in children under 5 years. Moreover, from the main causes of death for this demographic, it is the one with the lowest reduction between 2000 and 2013 (0.2%) (2); rather, the number of reported cases has increased during the last 2 decades. This not only urges for improved strategies to fight the disease, but it also questions our understanding of B. pertussis physiology.

Metabolic capabilities play a key role in defining allowable environmental niches of pathogens and therefore contribute to an understanding of the epidemiology of diseases. Metabolites also participate in host-pathogen interactions (3) and can provide new targets for therapeutic approaches. Yet, the understanding of the metabolism of B. pertussis has remained virtually unchanged since the 1960s, with only few recent studies dealing with this aspect of its physiology (for instance references 4 and 5). B. pertussis is described as fastidious in its nutritional requirements and is unable to survive outside the human host (6, 7). Today, it is still merely viewed as being unable to ferment sugars (8), is auxotrophic to niacin (9), and it strictly relies on amino acids as sources of energy, carbon, and nitrogen and on cysteine as a sulfur source (8). However, numerous discrepancies are found between the genome sequence information and the observed phenotypes, as well as between reports from different research groups. Some of the issues that we will study and resolve in this work, are (i) whether the tricarboxylic acid (TCA) cycle is fully functional (4, 5, 10), (ii) whether organic sulfur sources (cysteine) are mandatory for growth (11), and (iii) what are the missing nitrogen-containing end products of B. pertussis when growing *in vitro* on glutamate (several studies indicate a gap of up to 40% in the N balance [12, 13]).

Genome-scale models are inventories of the metabolic capabilities encoded in a genome. In recent years, powerful computational tools have been developed that allow the reconstruction of metabolic pathways for any organism (14, 15). These tools require genome information, basic cell physiology and biochemical knowledge, and some experimental data on cell growth. The reconstructed metabolic networks can subsequently be interrogated to define growth substrates and metabolic end products, as well as to identify links between parts of the metabolism that remain elusive to intuition (15).

In the present work, we used these techniques to reinvestigate the metabolism of B. pertussis from a systems-level perspective. In a first step, we developed a comprehensive genome-scale metabolic network for B. pertussis in an effort to reconcile physiological observations with genomic information. A detailed biomass reaction was formulated based on experimental data, and new pathways were introduced to accommodate the synthesis of biomass components. We further developed a novel algorithm that enumerates all possible minimal nutrient combinations that support the formation of this highly specific biomass. In this way, we could systematically probe the full metabolic capabilities of B. pertussis.

Through model-guided experimentation, we discovered that B. pertussis can be much more versatile than previously reported and is able to grow on very minimal media, where only the carbon source must be organic, although such behaviors are strain dependent. Our model also predicted the excretion of novel nitrogen-containing products that may shed new light on potential host-pathogen interactions. Finally, we used the validated model to rationally engineer new medium formulations for the production of vaccine antigens. Improved media resulted in up to 2.4-fold increased pertussis toxin production. Our work demonstrates the innovation potential of comprehensive *in silico* approaches but also highlights their limitations.

## RESULTS

### Metabolic network of B. pertussis Tohama I.

**FIG 1:**
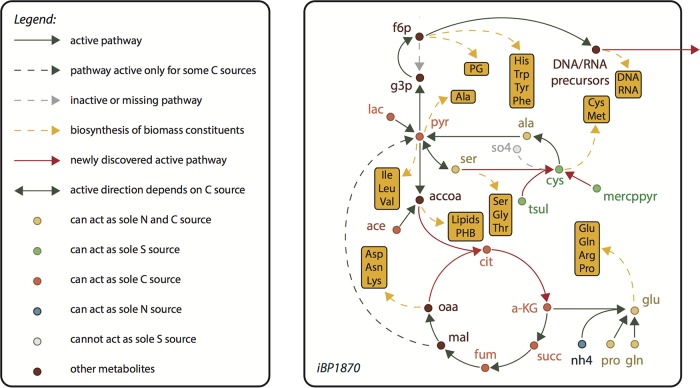
Genome-scale metabolic network of Bordetella pertussis Tohama I (iBP1870). Core metabolic network highlighting newly discovered active pathways and compounds that have been experimentally verified as possible sole sources of C, N, and/or S. PG, peptidoglycan; f6p, d-fructose 6-phosphate; g3p, glyceraldehyde 3-phosphate; pyr, pyruvate; lac, l-lactate; ser, l-serine; cys, l-cysteine; ala, l-alanine; so4, sulfate; mercppyr, mercaptopyruvate; tsul, thiosulfate; accoa, acetyl-coenzyme A; ace, acetate; cit, citrate; a-KG, α-ketoglutarate; succ, succinate; fum, fumarate; mal, malate; oaa, oxaloacetate; nh4, ammonium; pro, l-proline; gln, l-glutamine; glu, l-glutamate.

The metabolic network of B. pertussis Tohama I was reconstructed based on its genome sequence (16) using standard methods that rely on available genome-scale metabolic models (14, 17). Due to phylogenetic proximity, model iAF1260 for Escherichia coli MG1655 was used as the template (18) to generate an automated draft metabolic network for Tohama I. The draft network was extensively curated based on the literature and experimental data obtained in the present study. We started with a thorough experimental characterization of exometabolite fluxes (Fig. S1 and Data Set S1 in the supplemental material) and biomass composition (Data Set S2) at different growth stages, using reference batch fermentations in a chemically defined medium derived from the Stainer-Scholte medium (MSS-CDM). These data were used to calibrate and refine the model through iterative cycles of *in silico* simulations and experimentation. The final model was named iBP1870 (Fig. 1); it contains exchange reactions for 202 compounds defining the potential for uptake and excretion of compounds and 1,473 internal reactions, of which 1,017 are gene associated, representing 762 genes (22% of the genome) (Table S1).

Important features of the model include a detailed B. pertussis-specific biomass equation (Fig. S2) and many reactions not defined yet in the E. coli model, such as in pathways of amino acid metabolism, iron acquisition, sulfur metabolism, and biosynthesis of lipooligosaccharides and storage compounds (Data Set S3). In two pathways, a discrepancy was observed between sequence-based reconstruction and literature: (i) a fully functional TCA cycle was reconstructed (16), whereas the literature indicated this cycle is partially dysfunctional (10); and (ii) despite reported cysteine auxotrophy (12), genes encoding the full pathway from sulfate to cysteine are present, although the genes responsible for the early steps (conversion of sulfate into sulfite via 3′-phosphoadenylyl sulfate) are annotated as pseudogenes (16). To maintain consistency, the corresponding reactions were retained in the model but constrained to carry no flux by default, to account for their reported nonfunctionality (see the supplemental material).

Energy parameters were determined based on measured metabolic fluxes in reference fermentations, assuming a partially dysfunctional TCA cycle (no flux allowed from oxaloacetate to α-ketoglutarate [10]). The deduced biomass yield *Y*^*ATP*^ of 3.6 mg of dry cell weight (mg_DCW_) per mmol_ATP_ is in the range of previously reported values for B. pertussis (2.7 mg_DCW_ · mmol_ATP_^−1^; see reference 19 and Data Set S4). A value of 9.21 mmol_ATP_ · g_DCW_^−1^ · h^−1^ was calculated for the non-growth-associated maintenance energy requirement *m_ATP_*, which is in the range of values reported for E. coli (8.39 mmol_ATP_ · g_DCW_^−1^ · h^−1^; [18]) but higher than previously reported for B. pertussis (0.83 mmol_ATP_ · g_DCW_^−1^ · h^−1^; see reference 19 and Data Set S4). This discrepancy is likely attributable to the use of different strains and growth conditions (cultivation mode [batch versus chemostat], medium, pH regulation, and temperature).

**FIG 2:**
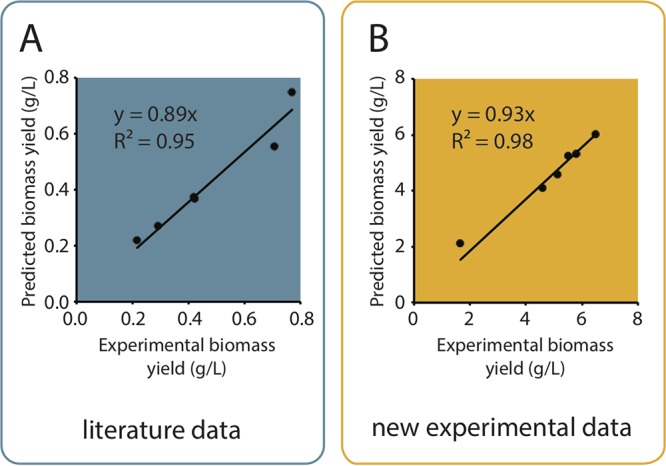
Validation of predicted biomass yields. (A) Validation versus experimental biomass yields in shake-flask cultures using media with various ratios of glutamate, lactate, and ammonia, as obtained from Thalen et al. (10). (B) Validation of predicted versus experimental biomass yields in pH-controlled batch fermentations using newly formulated media (Data Set S5). All simulations were made with a partially dysfunctional TCA cycle.

To validate the predictive value of the calibrated model, the growth yield of B. pertussis in 12 chemically defined media was simulated using flux balance analysis (FBA) with model iBP1870. Two data sets were used: (i) a set of 6 shake-flask cultures in media with various ratios of glutamate, lactate, and ammonia, published by Thalen et al. (10), and (ii) a set of 6 pH-controlled batch fermentations in newly formulated media, all containing l-Glu as a main source of carbon and nitrogen, with various additional N and C sources (Data Set S5). With both data sets, model predictions were in good agreement with experimentally measured yields, confirming the ability of the parameterized model iBP1870 to accurately and quantitatively predict biomass yields under various growth conditions (Fig. 2).

### Novel end products of N metabolism in B. pertussis.

The reference fermentations used for model calibration showed a massive apparent nitrogen imbalance, where ammonia and biomass accumulation only accounted for 37% and 32% of the N consumed via amino acid uptake, respectively. This left approximately 30% of the N unaccounted for. Such an imbalance had already been repeatedly observed (12, 13) but remains unexplained.

**FIG 3:**
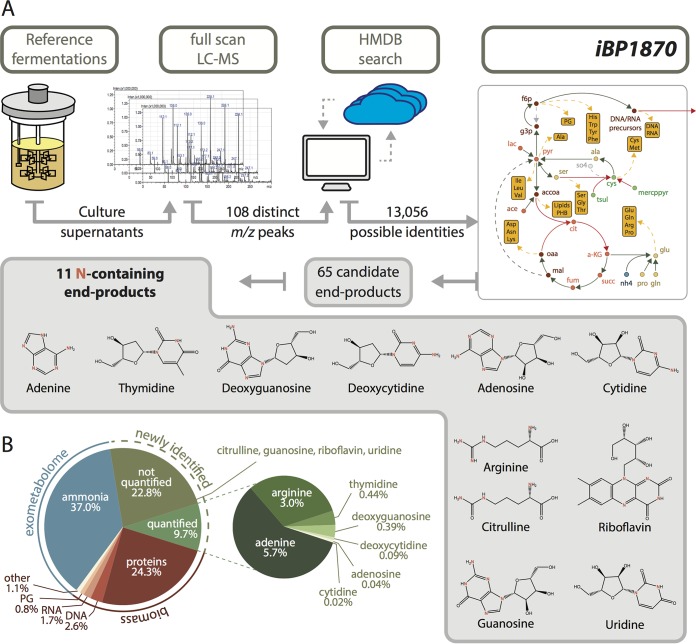
Novel end products of N metabolism in B. pertussis. (A) Schematic description of the procedure used for MS-based identification of metabolic end products. HMDB, human metabolome database; see the Fig. 1 legend for other abbreviations. (B) Overview of N sinks in reference fermentations highlighting newly identified sinks are in green.

The metabolic model was used to suggest additional N sinks: by constraining all measured input and output fluxes by their measured value, and given the mass balance constraints, all possible feasible flux values for unknown fluxes were computed by flux variability analysis (FVA) (20, 21). FVA predicted l-Arg, along with purine/pyrimidine nucleobases and their nucleoside/nucleotide derivatives, as potential N sinks. Using a liquid chromatography-mass spectrometry (LC-MS)-based approach (Fig. 3A), we were able to detect 11 additional N-containing end products, seven of which were further quantified over time in the reference fermentations used for model construction. In agreement with FVA predictions, arginine, adenine, adenosine, cytidine, deoxycytidine, deoxyguanosine, and thymidine were detected in increasing concentrations during the course of the fermentation (Fig. S3). Adenine alone accounted for 5 to 6% of the N balance. l-Arg represented an additional 3 to 6% (Fig. 3B).

The newly identified N sinks have a high N-to-C ratio (especially purines and arginine). Their secretion as an alternative to ammonia is therefore an efficient way to eliminate excess nitrogen while limiting the amount of carbon wasted. Nevertheless, such biosynthetic products are energy-costly “waste” products, as confirmed by the analysis of iBP1870, which indicated that the secretion of other compounds, such as alternative amino acids, would be preferable from an energetic perspective. The observed metabolic behavior therefore suggests a role for these alternative N sinks beyond mere nitrogen excretion.

### Minimal growth requirements of B. pertussis Tohama I.

**FIG 4:**
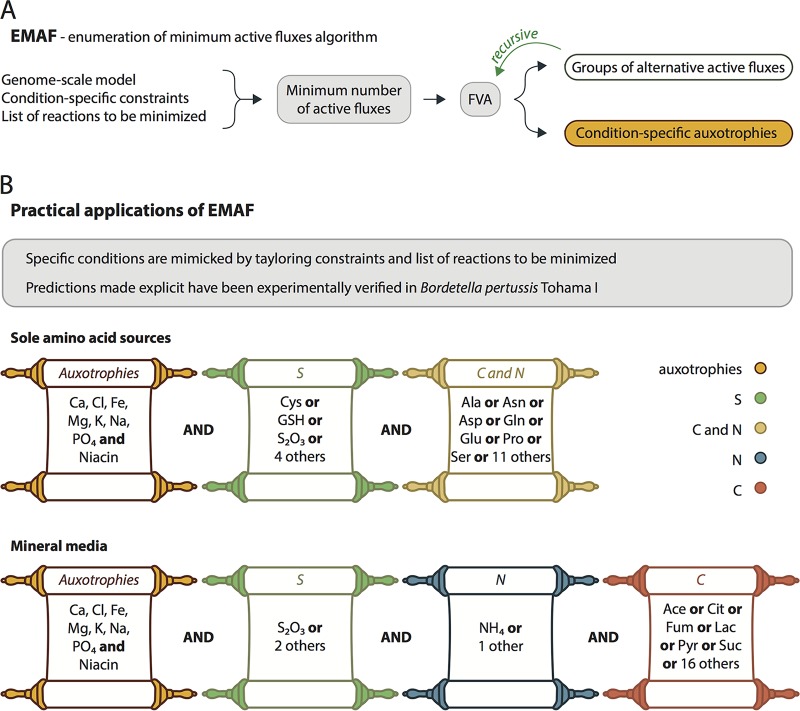
Enumeration of minimum number of active fluxes (EMAF) algorithm. (A) Schematic overview of EMAF. Based on the listed inputs, it enumerates all possible minimal combinations of active fluxes that satisfy a given criteria (detailed in supplemental material). (B) Practical applications of EMAF explored in this study. We minimized all input reactions and used different constraints to mimic specific conditions. All substrates depicted have been experimentally tested and confirm the metabolic versatility of B. pertussis.

To probe the full metabolic potential of B. pertussis Tohama I, we computed the combinations of minimal nutrient inputs that would support growth. As this is a combinatorial problem (the presence of one nutrient may affect the need for another), we developed enumeration of minimal active fluxes (EMAF) (Fig. 4 and S4), an algorithm that is able to (i) return the minimum number of active fluxes within a given set of reactions that support some objective flux, and (ii) enumerate all possible minimal combinations of fluxes (here, substrate uptake), specifically differentiating between absolutely required fluxes (auxotrophies) and sets of interchangeable fluxes (see the supplemental material). EMAF was implemented in the open-source modeling environment CBMPy (22).

**FIG 5:**
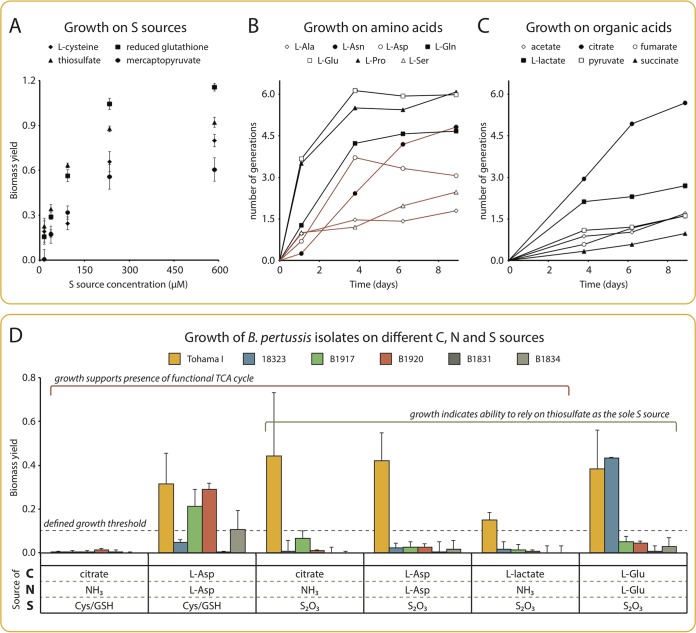
Minimal growth requirements of B. pertussis. (A) Growth of Tohama I with single S sources. Cells were grown in IMP-CDM lacking all S sources and supplemented with single S sources, as indicated, in 96-well microtiter plates. Biomass yield is expressed as the final OD, from which the final OD of the negative control (cultures in IMP-CDM with no S source) was subtracted. Each point represents the average of 4 independent repeats; error bars represent the standard deviation. (B and C) Growth of Tohama I with single amino acids as sole N and C sources (B) or single organic acids as sole C sources (C). Cells were cultivated in minimal media with no source of N and C, supplemented either with single amino acids (B) or with single organic acids and ammonia (C), in shake flasks. Growth is expressed as the number of generations from the start of the culture, from which the background number of generations was subtracted (negative control with no C source). (B) Black lines indicate conditions predicted not to require the TCA cycle, and brown lines indicate conditions predicted to require a fully functional TCA cycle. Each curve is from one representative experiment from at least 3 independent repeats. Growth was considered positive when cultures displayed at least one generation over the entire cultivation time. (D) Growth of alternative strains. Cells were cultivated in minimal media with different C, N, and S sources (as indicated), in 48-well plates. Biomass yield is expressed as the OD after 9 days, after deduction of the OD of cultures of the corresponding isolates in the same media but without a source of C. Data represent the average of at least 3 independent repeats; error bars represent the standard deviation. The growth threshold was arbitrarily set at a biomass yield of 0.1, which corresponds to approximately 1.6 generations from the start of cultivation. For all strains, positive controls in medium IMP-CDM confirmed growth (not shown).

We first applied EMAF to compute possible sulfur sources. B. pertussis is reported to be auxotrophic for cysteine, i.e., inorganic sulfur sources cannot support growth (11). In agreement, EMAF predicted no growth with l-Met, oxidized glutathione, sulfite, or sulfate as sole S sources, and it correctly predicted l-Cys and reduced glutathione as possible S sources in a minimal medium containing glutamate as the sole source of C and N. Unexpectedly, the algorithm also returned 5 additional S sources that would allow growth on the same minimal medium but had never been tested previously or had been reported to be nonfunctional, with 3 inorganic (sulfur dioxide, thiocyanate, and thiosulfate) and 2 organic (mercaptopyruvate and taurine) compounds. When tested experimentally, growth was confirmed with mercaptopyruvate and with thiosulfate (Fig. 5A), one of the most abundant inorganic sulfur species in the environment (23) and a constituent of human body fluids. According to the reconstituted metabolic network, thiosulfate is converted to cysteine in two steps: thiosulfate disproportionation (reaction associated with gene BP0431), yielding sulfite and hydrogen sulfide, followed by cysteine synthesis from *O*-acetylserine and hydrogen sulfide (cysteine synthase B, BP0958). This pathway allows the missing reactions to be bypassed in the biosynthesis of cysteine from sulfate. This is a significant finding, as it expands the range of possible S substrates from strictly organic to inorganic and non-cysteine-containing S sources.

B. pertussis is generally believed to require glutamate or proline exclusively for growth (11, 24), which is attributed to a partially dysfunctional TCA cycle (10). We used EMAF to probe the ability of B. pertussis Tohama I to grow on different amino acids as the sole C or N source in the presence of thiosulfate and oxygen. Without a complete TCA cycle, indeed, only glutamate and related amino acids (proline and glutamine) could sustain *in silico* growth. In contrast, with a complete TCA cycle, all amino acids but histidine (for which no catabolic pathway was identified) would suffice as the sole C or N source. Experimentally, the growth of strain Tohama I was observed with four amino acids that required a functional TCA cycle for *in silico* growth: aspartate and asparagine sustained growth to levels only slightly below those of glutamine, while serine and alanine resulted in modest, yet significant growth (Fig. 5B). Experiments did not match the *in silico* predictions for 10 amino acids (tyrosine and cysteine were not tested), which can likely be explained by regulatory limitations not considered in purely stoichiometric models (25).

One important consequence of the presence of a fully functional TCA cycle was the possibility to completely dissociate the N, S, and C sources as inorganic ammonia, inorganic thiosulfate, and an organic acid as the C source. These predictions challenged the paradigm that B. pertussis absolutely requires amino acids for growth and that organic acids can be used only in the presence of an amino acid source (10, 26). We confirmed that a number of organic acids, including citrate, l-lactate, and to a lower extent, acetate, pyruvate, fumarate, and succinate, were able to support growth in the absence of any amino acid (Fig. 5C), although the observed growth rates under such minimal conditions were very low.

These data provide compelling evidence for the presence of a complete TCA cycle, as predicted from the genome sequence of strain Tohama I. Altogether, these simulation-driven experiments indicate that the metabolic requirements of B. pertussis Tohama I are not only very minimal but also significantly more versatile than previously alleged. The minimal set of nutrients, apart from niacin (9) includes inorganic phosphate (P source), ammonia or amino acids (N source), thiosulfate, mercaptopyruvate, or cysteine-containing compounds (S source), and a source of C (amino acids or organic acids). Such nutrients are reasonably accessible not only in the human host but also in other non-host-associated ecosystems.

### Extrapolation to circulating Bordetella strains.

Strain Tohama I is a clinical isolate that was isolated before the vaccination campaigns, and its representativeness of the B. pertussis species has been questioned (27). We thus verified whether the demonstrated metabolic versatility of Tohama I would also apply to other strains of the Bordetella genus (B. pertussis, B. bronchiseptica, and B. parapertussis). Metabolic networks were reconstructed for 8 additional strains of B. pertussis, 4 strains of B. bronchiseptica, and 2 strains of B. parapertussis (16, 28–30) by curating the output of a newly developed web-based algorithm pipeline (http://f-a-m-e.org/fijo/) that facilitates the generation of genome-scale models based on genome sequence (Data Set S8). For each minimal medium composition that supported the *in silico* growth of B. pertussis Tohama I, the corresponding set of active reactions was retrieved from EMAF and compared to the reaction list of the reconstructed networks.

For all minimal substrate combinations that supported the growth of strain Tohama I experimentally, the necessary genes were also found in all other B. pertussis strains, irrespective of their geographical or temporal origin (Fig. S5). In order to validate these predictions experimentally, we selected 5 strains of B. pertussis, including strain 18323, the strain most distantly related to Tohama I (30), as well as 4 recent clinical isolates carrying either the *ptxP1* (B1834 and B1920) or *ptxP3* (B1831 and B1917) allele (29). These strains were cultivated in selected minimal media in order to validate the use of thiosulfate as a sulfur source and the functionality of the TCA cycle (Fig. 5D).

The presence of a functional TCA cycle was confirmed in 3 of the 4 recent isolates (B1917, B1920, and B1834), as demonstrated by their ability to grow with aspartate as a sole C or N source and an organic S source (cysteine and reduced glutathione). With this experimental setup, we could not demonstrate a functional TCA cycle in strains 18323 and B1831. Thiosulfate utilization was found to be restricted to strains Tohama I and 18323, as indicated by the absence of growth of the 4 recent isolates in a medium composed of glutamate as a sole C or N source and thiosulfate as a sole S source. Consistent with these results, only strain Tohama I was able to grow with TCA cycle-dependent C or C or N sources (aspartate, citrate, or l-lactate) when thiosulfate was the source of S. Intriguingly, whereas Tohama I was able to grow with citrate as a sole C source and thiosulfate as a source of S (Fig. 5D) (also see Fig. 5C and Table 1 for a confirmation of this behavior in alternative experimental setups), no growth was observed when thiosulfate was replaced with an organic source of S (cysteine and glutathione). Similarly, none of the 3 alternative strains shown to contain a functional TCA cycle could grow under these conditions.

**TABLE 1:** Growth and virulence factor production for B. pertussis strains Tohama I and 18323 cultivated in different chemically defined media^*a*^

Medium type and strain	Medium^*b*^	pH regulation	Initial OD_650_^*c*^	Final OD_650_	Fermentation duration^*d*^	Avg doubling time (h)^*e*^	PT yield (mg/liter)^*f*^	FHA yield (mg/liter)^*g*^
Minimal media								
Tohama I	SS	50% (wt/vol) H_3_PO_4_	0.138	1.8	63h00	17.2	0.8 ± 0.4	10.5
	CIT-NH3	50% (wt/vol) H_3_PO_4_ and 1 M K_2_HPO_4_	0.007	6.5	159h00	16.1	<0.5	27.4
Rich media								
Tohama I	MSS-CDM^*f*^	50% (wt/vol) acetic acid	0.168	8.7	45h40	7.0	15.2 ± 1.3	124.9
	IMP-CDM	50% (wt/vol) H_3_PO_4_	0.155	8.3	47h00	7.9	29.0 ± 2.6	ND
	IMP2-CDM	50% (wt/vol) H_3_PO_4_	0.177	8.3	41h15	7.2	36.7 ± 2.2	103.2
	LCMSSB	50% (wt/vol) H_3_PO_4_	0.104	4.1	33h00	6.2	2.7 ± 1.8	14.1
	MSS-CDM	50% (wt/vol) H_3_PO_4_	0.159	7.8	39h00	6.7	22.5 ± 3.9	112.9
	IMP2-CDM-AA as in MSS-CDM	50% (wt/vol) H_3_PO_4_	0.167	8.7	43h15	7.6	32.3 ± 1.7	ND
18323	MSS-CDM	50% (wt/vol) acetic acid	0.040	8.1	51h00	6.7	4.7 ± 2.2	10.0
	IMP2-CDM	50% (wt/vol) H_3_PO_4_	0.023	10.1	74h00	8.5	17.3 ± 2.6	34.0

a All fermentations were performed with a chemical antifoam for foam control.

b Medium compositions provided in Table 3.

c Initial biomass concentration calculated based on measured OD_650_ of the preculture and inoculum/medium volumes.

d The total fermentation time is defined as the time at which oxygen consumption decreases (as a consequence of glutamate exhaustion), resulting in a decrease in stirring speed.

e Average generation time calculated as the ratio between OD_650_ at the end of fermentation and OD_650_ at the start of fermentation, converted to log_2_, and divided by the total fermentation time.

f Average of at least four independent repeats of the PT ELISA ± standard deviation.

g Average of two independent repeats. ND, not determined.

In conclusion, we were able to demonstrate that some of the newly identified metabolic features observed in Tohama I extend to other B. pertussis strains (including recent clinical isolates). However, we cannot conclude that this is a general characteristic of the species, as only Tohama I appears to combine a functional TCA cycle and the ability to use thiosulfate. The discrepancy between experimental observations and genome sequence information can possibly be explained by a differential regulation of gene expression between strains, a phenomenon recently shown to affect not only virulence genes but also metabolic genes in B. pertussis (31). Posttranscriptional layers of regulation might also play a role. The observation that the functionality of the Tohama I TCA cycle is obscured under certain conditions further supports the role of regulation in this context. It thus seems that the metabolic flexibility of B. pertussis is a strain-dependent trait governed by regulation rather than mere gene content.

### New medium formulations for vaccine production.

The new metabolic knowledge gained from our model-based approach is contained within the genome-scale model (Model S1, available at https://github.com/SystemsBioinformatics/pub-data/tree/master/bordetella-pertussis-model), both from a qualitative (new functional pathways) and quantitative (energy parameters) point of view. A straightforward application of such information is the optimization of growth media. We thus tested whether improved fermentation media could be designed for vaccine production, based on this newly acquired knowledge.

For pertussis vaccines, whether whole cell or acellular, the fermentation process is critical, as it is the step where antigens are produced. Yet, current industrial culture conditions of B. pertussis result in relatively poor yields of the different antigens, more specifically pertussis toxin (PT) (32). PT production in minimal media, such as the classical Stainer-Scholte (SS) medium, is low in the absence of additional amino acids (4). This was confirmed in a 20-liter scale-down model of an industrial fermentation process with strain Tohama I (Table 1). Based on the discovered metabolic capabilities of B. pertussis Tohama I, we tested a minimal medium containing only citrate as a C source and ammonia as an N source (CIT-NH3). Significant growth was observed (biomass yield over 3-fold higher than with SS medium; Table 1), confirming the functionality of the TCA cycle of B. pertussis Tohama I under industrial-like conditions. However, growth was slow and resulted in extremely low antigen production (especially PT [Table 1]), making such amino acid-free media unsuitable for industrial vaccine production.

Since minimal media based on alternative substrates did not improve PT production, we subsequently aimed to rationally improve MSS-CDM, a medium that contains 14 amino acids in addition to glutamate and that already produces acceptable levels of PT (Table 1). We focused on PT as a proxy for the global antigen production, as PT belongs to the most tightly regulated class of virulence factors in B. pertussis, and conditions that result in high PT production also promote high-level expression of all other virulence factors (33, 34). Model iBP1870 does not contain PT explicitly; yet, assuming PT and biomass production are coupled, optimization of the basic MSS-CDM process was performed using FBA, with biomass as the objective. We applied as constraints that it must: (i) produce the exact same biomass yield as that obtained with the original basic MSS-CDM; (ii) exclude sulfate as a substrate, as sulfate is one of the most potent inhibitors of PT production (35); (iii) allow (not force) all components of the basic MSS-CDM to be taken up at maximally twice the amount actually consumed in the reference fermentations; and (iv) not restrict end product formation except for sulfate, sulfite, and sulfur dioxide, compounds known to inhibit PT production (modulators of the Bvg two-component transduction system, which regulates virulence gene expression). As an output, a list of optimal balanced ratios between substrates was obtained.

The predicted medium (IMP-CDM) was tested experimentally with strain Tohama I and found to support a growth yield and rate similar to those of the initial MSS-CDM, with a 1.9-fold higher production of PT (Table 1). By repeating the FBA-based procedure and allowing alternative S sources as the substrates, a medium containing thiosulfate instead of cysteine was designed (IMP2-CDM), which resulted in a 2.4-fold improvement of PT production compared to that with the reference MSS-CDM process (Table 1). This represents a 14-fold increase compared to PT production by Tohama I when grown according to the best batch process published to date (medium LCMSSB [36]; Table 1). Filamentous hemagglutinin (FHA) production remained unaffected in IMP2-CDM compared to the reference MSS-CDM conditions (Table 1). We found a similar relative improvement of PT yield with the distantly related strain 18323 (Table 1).

Model-driven process and medium modifications accounted for 91% of the total PT improvement of Tohama I grown in IMP2-CDM, as estimated from fermentations in which specific subsets of modifications were not implemented (Table 2). The highest contributor to PT yield improvement was the replacement of cysteine with thiosulfate (36%), a direct output of FBA when imposing the constraint that no sulfate can be produced and that S sources other than cysteine are allowed. Thiosulfate was found to be as potent as sulfate as a modulator of PT production (data not shown). However, thiosulfate serves here as an S source and is therefore consumed during growth in the optimized medium, which gradually relieves BvgAS-mediated repression of PT production; conversely, in classical media using cysteine as a source of S, sulfate progressively accumulates as a result of cysteine catabolism, resulting in a steadily decreasing PT production during the course of the fermentation process (36). Equally important (34% of all improvements) was the replacement of acetic acid with phosphoric acid for pH regulation, which results in lower acetate accumulation (an inhibitor of growth and macromolecule synthesis [37]) and higher phosphate supply. This process modification was introduced after the detailed metabolic characterization of the reference fermentations, in which acetate accumulated up to 180 mM but phosphate was limiting (Fig. S1).

**TABLE 2:** Quantitative contribution of medium and process changes to increased PT yield with IMP2-CDM-based process

Change description	Relative PT yield^*a*^	Relative contribution to overall PT yield improvement (%)
Replacement of cysteine with thiosulfate as S source	1.51^*b*^	34
pH control with phosphoric instead of acetic acid	1.48^*c*^	36
Balancing of amino acid concn	1.29^*d*^	21
Total^*e*^	2.28	91
Unexplained/other changes^*f*^	1.13	9

a Compared to the reference process using medium MSS-CDM and acetic acid for pH regulation (Table 1).

b Calculated as the difference between the relative PT yield with IMP2-CDM (thiosulfate) and IMP-CDM (cysteine) media, compared to MSS-CDM medium with acetic acid for pH regulation (Table 1).

c Relative PT yield of fermentation using phosphoric acid or acetic acid for pH regulation with MSS-CDM medium (Table 1).

d Calculated as the difference between the relative PT yield with IMP2-CDM (balanced amino acid concentrations) and IMP2-CDM-AA as in MSS-CDM (original amino acid concentrations) media, compared to MSS-CDM medium with acetic acid for pH regulation (Table 1).

e Total improvement that can be explained with the above-listed medium and process changes.

f The unexplained part of PT yield improvement can possibly be attributed to other medium changes in IMP2-CDM versus MSS-CDM (buffer, additional minerals, and/or additional vitamins), although each of these, when tested separately, did not show a significant effect on PT yield (data not shown).

The third major contributor to PT improvement was the balancing of amino acid concentrations, which accounted for 21% of the PT yield increase in the improved medium (Table 2). The reason why amino acid concentrations have such a significant effect on PT production is unclear. Although it cannot be excluded that this effect is the consequence of changing the concentration of one (or a few) specific amino acid(s) with regulatory function, it may also reflect a globally more balanced, and hence, energetically efficient, cometabolism of substrates. This is supported by the higher ammonia production under improved cultivation conditions, representing 52% of the N end products (only 37% in the basic MSS-CDM process). Such a precise balancing of substrate concentrations can only be achieved by taking into account the complex metabolic interconnections between pathways at the whole-cell level, combined with a fine knowledge of biomass composition. In this context, genome-scale mechanistic models provide a clear benefit over traditional optimization techniques based on empirical correlations between parameters, as the traditional techniques are virtually unable to provide balanced media, and they require a very high number of cultures.

## DISCUSSION

In this work, we reconstructed a comprehensive genome-scale metabolic network for Bordetella pertussis. Model iBP1870 is much more detailed than the only B. pertussis model in the literature, which was limited to reactions involving amino acids and by-products considered to be main contributors to the carbon and nitrogen balance (4). iBP1870 is the result of multiple cycles of manual curation, in which the network was tuned to match genome sequence information of strain Tohama I, literature data, and newly generated experimental evidence. A particular effort was invested in defining the macromolecular composition of the cells; this resulted in insights into previously undescribed pathways or metabolites. iBP1870 includes detailed biosynthetic pathways for B. pertussis-specific metabolites, among which are lipooligosaccharides (LOS) and the siderophore alcaligin, two key virulence factors of this pathogen (6); it also includes more general pathways for polyphosphate and amino acid metabolism, for which 84 reactions were added (Data Set S3). The resulting model is thus a knowledge base that will be a valuable resource for future metabolic reconstructions of related strains and organisms.

In the present study, we illustrated the use of the B. pertussis model in contexts relevant to the overall physiology and ecology of this organism, as well as to biotechnological applications.

First, we explored the metabolic landscape in terms of nutrient requirements and identified a greatly expanded repertoire of growth modes for B. pertussis Tohama I. Importantly, a significant proportion of these novel growth regimes require a key pathway (the TCA cycle) whose functionality in B. pertussis was subject to debate (4, 5, 10). Although two recent studies reported a similar conclusion in strains Tohama I (5) and 10536 (4), their evidences were based on the observation that metabolic flux distributions were better accounted for from a quantitative perspective, when assuming this pathway was functional. Nevertheless, growth could also be explained qualitatively without a TCA cycle, under their conditions. Izac et al. also demonstrated that the necessary genes were transcribed and that the corresponding enzymatic activities were detectable in *in vitro* assays (5). Here, by demonstrating growth on a number of organic acids as sole C sources, we provide direct *in vivo* evidence that the TCA cycle of strain Tohama I is fully functional: with organic acids, such as citrate, as the sole C source, growth cannot be explained without a TCA cycle. The functionality of the TCA cycle, however, appears to be under the control of regulatory networks, making it a strain-dependent characteristic: it could only be demonstrated experimentally with 3 of the 5 alternative strains tested, despite the presence of the necessary genes in all genomes.

Cysteine auxotrophy, as another major paradigm of pertussis metabolism (11, 16), was also contradicted by model simulations and experimental evidence: B. pertussis Tohama I is able to use a significantly wider range of S sources than generally accepted (including S-containing organic acids and inorganic thiosulfate), which demonstrates the functionality of new pathways for the *de novo* synthesis of cysteine. This metabolic trait was only confirmed in one additional strain (18323), whereas 4 recent clinical isolates failed to grow experimentally with thiosulfate as a sole S source, despite the presence of the genes required for thiosulfate utilization.

Altogether, our findings thus reveal three key features of B. pertussis metabolism. First, it can be versatile, as characterized by the large variety of substrate combinations that can be used for growth. Second, it can be minimal, in the sense that growth is possible in minimal, nearly mineral media (only the C source must be organic). Third, the above-mentioned characteristics seem, however, to rely on regulatory (nonmetabolic) strain-specific features, resulting in strain-to-strain variability. This likely explains discrepancies between different reports, such as the debate about the presence of a functional TCA cycle (4, 5, 10).

Although the demonstrated metabolic versatility of B. pertussis appears to vary between strains (Tohama I being the only case where both the TCA cycle and the thiosulfate utilization pathways are functional), our results indicate that the general acceptation that B. pertussis is fastidious might be too restrictive. Even if regulatory constraints or mutations make certain functions inactive in other strains, the presence of the necessary structural genes is conserved, providing potential for evolution or for expression under yet-unidentified conditions. Metabolic flexibility could participate in the survival of B. pertussis in the host, but also in external environments, although other factors, such as the known susceptibility of B. pertussis to fatty acids (38) or to abiotic factors (temperature and humidity), probably also play a key role in this context.

As a second application example, we used the B. pertussis metabolic model as a guide to identify novel end products of nitrogen metabolism. We demonstrated the production of significant amounts of purines, purine nucleosides, and arginine as N sinks (Fig. 3B). Although it cannot be excluded that this observation is the consequence of artificial growth conditions that differ from the natural environment of B. pertussis, such an energetically unfavorable metabolic pattern might also reflect a biological role for these compounds beyond N excretion. To date, the study of interactions between B. pertussis and its human host have concentrated mostly on protein-based toxins and adhesins (for example, see reference 6) despite the role of metabolic interactions in this context (3). Purine nucleosides are well-known modulators of innate immunity, for example, by decreasing the synthesis of the antimicrobial nitric oxide (NO) through a diversity of mechanisms (39). Accordingly, adenosine production and a functional purine biosynthetic pathway have been identified as key virulence factors in several pathogens (40–44). Arginine production by B. pertussis could further inhibit NO production by favoring the accumulation of asymmetric dimethyl-arginine, an endogenous inhibitor of NO synthases (45, 46). The roles of NO and arginine in respiratory diseases, such as asthma or cystic fibrosis, which share several symptoms with whooping cough, are well established (45, 46). Therefore, we speculate that the combined production of arginine and purine nucleosides contributes to the virulence of B. pertussis by subverting the host innate immunity: arginine and purine production might thus constitute alternative targets against B. pertussis infection.

Finally, we also demonstrated the use of the metabolic model for rational medium and bioprocess design in the context of industrial pertussis vaccine production. Using an approach based on FBA and the metabolic knowledge acquired during model construction, a medium was designed that increased the production of PT (the limiting antigen in acellular pertussis vaccine manufacturing [32]) by 2.4-fold. Alternative approaches for increasing antigen yields in B. pertussis usually involve genetic engineering (32, 47), with significant regulatory implications for drug approval. The improved medium and process conditions developed in this study are readily applicable to current vaccine strains, such as Tohama I, but also apply to more distantly related strains, such as 18323 (Table 1). They could also be used in combination with genetic engineering to further increase yields. From a drug approval perspective, the proposed modifications are based on a mechanistic understanding of metabolism and regulation of virulence factors in B. pertussis, providing a sound scientific rationale for process change. Such a model-guided rational process improvement strategy is thus perfectly aligned with quality-by-design principles.

### Concluding remarks.

**FIG 6:**
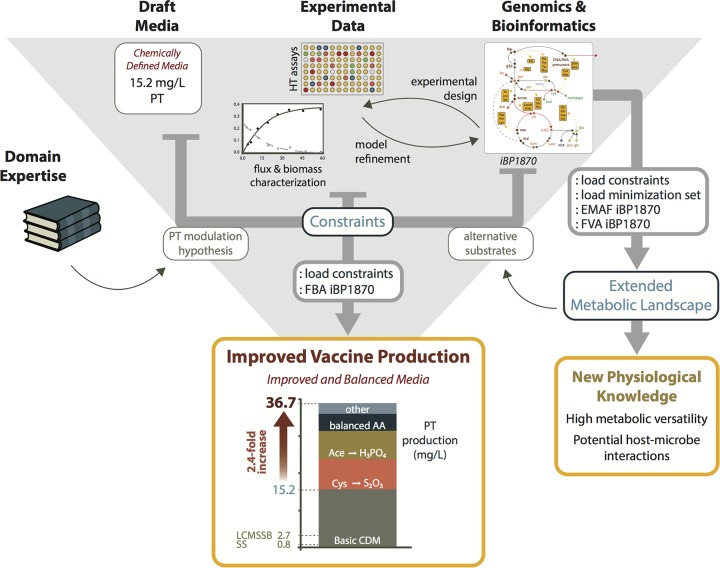
Model-based strategy to improve medium composition and generate new physiological knowledge. Arrows represent information flow. HT assays, high-throughput growth assays.

The approach taken here to probe the full metabolic potential of an organism is general and can be applied to any sequenced organism for which a minimum amount of experimental data are accessible or can be generated (Fig. 6). Here, we used flux data from only two fermentations to adequately calibrate our model. We show how subsequent model-guided experimentation can provide progress in metabolic understanding of the physiology of a poorly characterized organism. In this way, we redefined the physiology and metabolic potential of B. pertussis: it is much more versatile than previously reported, although this appears to depend on regulatory strain-specific determinants. This study thus highlights the potential, but also limitations, of models solely based on metabolic gene content. Deciphering and taking into account the mechanisms responsible for the regulation of metabolic capabilities therefore appear to be crucial for a comprehensive understanding of the lifestyle of B. pertussis and the epidemiology of whooping cough.

## MATERIALS AND METHODS

### Reference fermentations.

Two batch fermentations of B. pertussis Tohama I (fermentations A and B) were performed in modified Stained-Scholte medium (MSS-CDM) (Table 3). The preculture consisted of 3 serial shake-flask cultures in MSS-CDM that were incubated at 35°C under constant orbital agitation (150 rpm) for approximately 24 h and harvested in exponential phase. The third preculture (1.5 liters; optical density at 650 nm [OD_650_] between 1.5 and 1.6) was used to inoculate a 20-liter fermentation vessel (Biolafitte) containing 10 liters of MSS-CDM. The fermenter was operated in batch mode at constant temperature (35°C) and head pressure (40,000 Pa). The culture was sparged with air at a constant rate of 20 liters/min. A mechanical foam breaker was used to control foaming. pH was regulated at 7.2 by automatic addition of 50% (wt/vol) acetic acid. The level of dissolved oxygen was regulated at 25% of its initial level by modulating the stirring speed. Sampling was performed at the start of fermentation and every 2 h between 18 h and the end of fermentation (abrupt decrease in oxygen demand). Supernatants and cells were separated by centrifugation (15,000 × *g*, 15 min, 4°C) and stored at −20°C. Biomass concentration was monitored as the OD_650_ and dry cell weight (DCW).

**TABLE 3:** Fermentation medium composition^*a*^

Compound	Concn (mg/liter)				
	MSS-CDM	CIT-NH3	IMP-CDM	IMP2-CDM	IMP2-CDM-AA as in MSS-CDM
l-Proline	1,040	0	882	882	1,040
Na-l-glutamate	20,000	0	18,677	18,677	18,677
l-Cysteine HCl	40	0	4	0	0
Na_2_S_2_O_3_·5H_2_O	0	5.65	0	2.83	2.83
NaCl	2,500	0	73	73	73
KH_2_PO_4_	500	500	500	500	500
KCl	200	200	200	200	200
MgCl_2_·6H_2_O	100	1,000	1,000	1,000	1,000
CaCl_2_·2H_2_O	20	20	20	20	20
FeSO_4_·7H_2_O	10	0	0	0	0
Fe(III)-citrate·3H_2_O	0	20	20	20	20
Tris	6,100	0	0	0	0
CuCl_2_·2H_2_O	0	1.28	1.28	1.28	1.28
CoCl_2_·6H_2_O	0	0.42	0.42	0.42	0.42
ZnCl_2_	0	10	10	10	10
MOPS	0	2,500	2,500	2,500	2,500
Ascorbic acid	400	623	623	623	623
Reduced glutathione	150	233	233	233	233
Niacin	4	6	6	6	6
Dimethyl-β-cyclodextrin	1,000	1,000	1,000	1,000	1,000
Na acetate	0	0	409	409	409
l-Alanine	312	0	304	304	312
l-Aspartic acid	436	0	524	524	436
l-Glutamic acid	1,600	0	3,475	3,475	3,475
l-Histidine	188	0	32	32	188
l-Glycine	163	0	149	149	163
l-Isoleucine	288	0	244	244	288
l-Leucine	484	0	438	438	484
l-Lysine HCl	600	0	393	393	600
l-Methionine	156	0	116	116	156
l-Phenylalanine	250	0	234	234	250
l-Serine	230	0	187	187	230
l-Tyrosine	67	0	34	34	67
l-Valine	456	0	399	399	456
Thiamine-HCl	0	10	10	10	10
Biotin	0	0.2	0.2	0.2	0.2
Riboflavin	0	0.3	0.3	0.3	0.3
Calcium pantothenate	0	4	4	4	4
Citric acid monohydrate	0	26,268	0	0	0
Ammonium hydroxide 25%	0	5,066	0	0	0

a pH adjustment was made to pH 7.4 (25°C) with 5 M NaOH and 6 M HCl.

### PT and FHA quantification.

PT and FHA concentrations were determined by enzyme-linked immunosorbent assay (ELISA) in the culture supernatants, as previously described (48).

### Determination of exometabolite fluxes in reference fermentations.

In addition to biomass and PT, a total of 37 metabolites were assayed (Data Set S1). The conversion of exometabolite concentrations to net fluxes is described in details in Data Set S1. Briefly, exometabolite concentrations were converted to absolute amounts (millimoles for metabolites and grams [dry cell weight] [g_DCW_] for biomass) and corrected for water evaporation, acetic acid addition, and sampling. For each metabolite (including biomass), the net production/consumption was calculated at every time point by subtracting the amount initially present from the amount at the time point considered (symbols in Fig. S1). The time courses of net production/consumption of each metabolite were then spline-fitted using the method of Klasson (49), with a smoothness factor of 3 (solid and dotted lines in Fig. S1). Phases of constant metabolism were determined from the spline functions (vertical solid lines in Fig. S1, and Data Set S1).

### Determination of biomass composition.

The macromolecular composition of cells was determined based on three sources: (i) experimental measurements at different growth stages on cells issued from the two batch reference fermentations (DNA content, RNA content, protein content, and amino acid composition, inorganic phosphate, and polyphosphate), (ii) literature data, and (iii) theoretical calculations (Data Set S2). Growth was modeled as a sink of biomass components in a proportion that matches biomass composition (Fig. S2).

### Model construction.

The metabolic network of B. pertussis Tohama I was reconstructed in three phases: (i) semiautomatic reconstruction based on model iAF1260 of E. coli MG1655 (18) using AUTOGRAPH (50), (ii) the addition of new reactions to explain experimentally observed metabolic behaviors in reference fermentations, and (iii) final manual curation and incorporation of default constraints to account for reported physiological observations. Model construction is described in details in the supplemental material and Data Set S3. The curated model iBP1870 is provided in SBML format at our GitHub repository: https://github.com/SystemsBioinformatics/pub-data/tree/master/bordetella-pertussis-model.

### Determination of energy parameters.

Maintenance and growth-associated ATP consumption were determined using flux balance analysis (FBA) on model iBP1870, as previously described (51), using extracellular flux constraints derived from the two reference batch fermentations (each subdivided into 5 phases of constant metabolism). The mathematical framework and assumptions underlying the calculations are described in details in the supplemental material and Data Set S4.

### Model validation.

FBA was used in combination with model iBP1870 to compute the maximum possible biomass production under constraints reflecting substrate availability in different media (Data Set S5). For every simulation, maintenance energy consumption was calculated based on measured growth parameters (initial biomass, cultivation time, and specific growth rate) and was introduced in the simulations as the lower bound of the R_ATPM ATP hydrolysis reaction (Data Set S5). Additional constraints were set to mimic a partially nonfunctional TCA cycle. The simulation results were compared to experimental growth yields, as derived from the literature (10) (data of Fig. 2A) or as generated by cultivating strain Tohama I in newly formulated media (Data Set S5) under the same conditions as in the reference fermentations (Fig. 2B).

### LC-MS analyses of reference fermentations.

For the qualitative identification of N sinks, supernatant samples from the endpoint of the two reference fermentations were mixed (1:1 ratio), diluted (1.7-, 3.3-, 5.5-, or 10-fold), and analyzed by LC-MS, as described in the details in Data Set S6. We found 108 *m*/*z* values (88 in positive mode, 20 in negative mode) whose peak areas varied proportionally between at least two consecutive dilutions, corresponding to 13,056 possible matches in the human metabolome database (52) (tolerance set to less than 0.1 Da). Of these, 65 compounds were also found in the metabolic network of B. pertussis, 32 of which were analyzed by LC-tandem MS (LC-MS/MS) to confirm their presence in the fermentation supernatants by comparison with commercial standards: a compound was considered present in a sample provided (i) the retention time (RT) did not deviate by more than 0.5 min compared to the RT of the standard (pure compound) and (ii) at least 2 of the characteristic MS/MS transitions (as determined with the standards) were detectable. Among the 12 metabolites confirmed to be present at the end of the reference fermentations (including 11 N-containing end products), six (adenine, adenosine, cytidine, thymidine, deoxyguanosine, and deoxycytidine) were quantified at different time points of the fermentations, by standard addition using LC-MS/MS (Data Set S6). Adenine was also quantified with an enzyme-based assay (53). l-Arginine was quantified with an enzymatic kit (K-Large; Megazyme, Ireland).

### Prediction of minimal growth requirements.

For strain Tohama I, the EMAF algorithm (supplemental material) was used in combination with model iBP1870 and appropriate constraint sets (Data Set S7) to screen single S, C or N (amino acids), and C sources (organic acids) that can support growth of B. pertussis Tohama I *in silico* with or without a functional TCA cycle.

For the 14 alternative Bordetella strains (30), the metabolic networks were reconstructed with FiJo (http://f-a-m-e.org/fijo) using model iBP1870 as the template, and manually curated (Data Set S8). From the metabolic flux distribution for each minimal medium composition that did support *in silico* growth of B. pertussis Tohama I (Data Set S7), the set of active reactions was extracted. Growth was determined to be feasible if all required reactions for *in silico* growth of B. pertussis Tohama I were also present in the reconstructed network; conversely, if only one required reaction was not present, growth was assumed to be not feasible.

### Experimental validation of minimal growth requirements.

Experimental validation of S sources with Tohama I was done with IMP-CDM medium (Table 3) lacking l-cysteine, l-methionine, and glutathione and supplemented with sodium thiosulfate, reduced glutathione, l-cysteine–HCl, or mercaptopyruvate at different concentrations, or with water as a negative control. Growth assays were performed in 96-well plates (Greiner 655090) containing 180 μl of medium and 20 μl of inoculum. The inoculum was prepared by growing cells in MSS-CDM in a shake flask to an OD_650_ between 1 and 2, collecting cells by centrifugation (5,000 × *g*, 15 min, 25°C), washing the cell pellet twice with 1 volume of 0.9% (wt/vol) NaCl, and resuspending cells in 0.9% (wt/vol) NaCl at a theoretical OD_650_ of 0.5. After inoculation, the 96-well plate was incubated for 7 days at 35°C and constant double orbital agitation in a Synergy H1 microplate reader (BioTek). Growth was monitored every 10 min as the OD_650_.

For all other validations, the inoculum was prepared as described above (except for resuspension of the washed cells [see below]), and growth assays were performed with a basal medium containing no source of C, N, or S (0.5 g/liter KH_2_PO_4_, 2.5 g/liter morpholinepropanesulfonic acid [MOPS], 0.2 g/liter KCl, 6 mg/liter niacin, 1 g/liter dimethyl-β-cyclodextrin, mineral salts [1 g/liter MgCl_2_·6H_2_O, 100 mg/liter CaCl_2_·2H_2_O, 20 mg/liter Fe(III)-citrate·H_2_O, 1.28 mg/liter CuCl_2_·2H_2_O, 0.42 mg/liter CoCl_2_·6H_2_O, 10 mg/liter ZnCl_2_]) and vitamins (0.2 mg/liter biotin, 0.3 mg/liter riboflavin, 10 mg/liter thiamine-HCl, 4 mg/liter calcium pantothenate), to which sources of C, N, and S were added as required. Media were adjusted to pH 7.4 (25°C) by addition of 5 M NaOH or 6 M HCl; the concentrations of Na^+^ and Cl^−^ were adjusted to at least 110 mM and 30 mM, respectively, with NaCl.

For the validation of amino acids as sole C or N sources and organic acids as sole C sources with Tohama I, each medium contained thiosulfate 0.25 mM (S source) and either a single amino acid as the sole C or N source (125 mM, except l-Asp (30 mM) and l-Trp (40 mM) or a single organic acid (100 mM acetate, pyruvate, citrate, l-lactate, succinate, or fumarate) as the sole C source, together with NH_4_Cl (25 mM) as the sole N source. As a negative control, a medium containing ammonia but no C source was used. Growth assays were performed in 250-ml shake flasks containing 30 ml of medium and inoculated with 1 ml of a washed cell suspension (OD_650_, 1.2) of exponentially growing B. pertussis Tohama I. After inoculation, cultures were incubated for 9 days at 35°C under constant orbital agitation (150 rpm). Growth was monitored offline as the OD_650_.

For validations with alternative strains (B1831, B1834, B1917, B1920, and 18323, as well as Tohama I as a control), the following C, N, and S sources were added. The S source was either sodium thiosulfate (62 mg/liter) or a mixture of l-cysteine (40 mg/liter), reduced glutathione (233 mg/liter), and ascorbic acid (623 mg/liter). For organic acids as C sources, 1.337 g/liter NH_4_Cl was added as an N source, together with 21.014 g/liter citric acid monohydrate or 21.005 ml/liter of a 40% l-lactic acid solution. For amino acids as C or N sources, l-aspartate (4 g/liter) or a mixture of sodium l-glutamate monohydrate (21.79 g/liter) and l-glutamic acid (1.579 g/liter) was used. As negative controls, strains were grown in media containing the same S source as the test cultures and ammonia as an N source but no C source. As positive controls, the 6 strains were grown in IMP-CDM. Growth assays were performed in FlowerPlate 48-well microtiter plates (m2p labs), with each well containing 800 μl of culture (720 μl of medium and 80 μl of inoculum, consisting of a washed cell suspension [OD_650_, 0.5] of exponentially growing cells). After inoculation, plates were covered with a Breathe-Easy membrane (Sigma) and pierced with 3 holes per well, and incubation was performed in a BioLector microbioreactor (m2p labs) at 35°C under constant agitation (800 rpm). After 9 days, the OD_650_ was measured offline.

### Model-based design of optimized media.

Optimization of the basic MSS-CDM was performed using FBA, with constraints as indicated in Results (“New medium formulations for vaccine production”). FBA outputs were converted into medium formulations (IMP-CDM and IMP2-CDM) based on an l-glutamate concentration of 120 mM. Additional modifications of medium composition (buffer, vitamins, and inorganic ions; not deduced from FBA) were as follows (Table 1): niacin, reduced glutathione, and ascorbic concentrations increased 1.5-fold; four additional vitamins (riboflavin, thiamine, pantothenate, and biotin) were added; three additional inorganic ions (Cu^2+^, Co^2+^, and Zn^2+^) were added; Mg^2+^ concentration increased 10-fold; iron was provided as Fe(III)-citrate instead of FeSO_4_; and Tris buffer was replaced with MOPS.

### Additional fermentations for evaluation of optimized media.

Optimized media were tested in 20-liter scale fermentations performed under the same conditions as the reference fermentations, except for (i) medium composition (Table 3), (ii) strain (Table 1), (iii) a foam control (mechanical foam breaker replaced with a chemical antifoam, as a 2-fold dilution of 30% simethicone [Dow Corning] in water), and (iv) pH regulation (Table 1). For fermentations in SS medium, the formulation described in Bogdan et al. (36) was used. For fermentations with LCMSSB, the conditions of Bogdan et al. (36) were used, which differ from the conditions of other fermentations in the following aspects: (i) the third preculture was performed in LCMSSB medium (supplemented with 1 g/liter dimethyl-β-cyclodextrin), (ii) the 20-liter-scale fermentation was performed in 11 liters of LCMSSB medium inoculated with 1 liter of a preculture (OD_650_, 1.25), (iii) when the culture reached an OD_650_ of 3, amino acid supplements (see composition in Bogdan et al. [36]) were added to the fermentation, together with a supplement of FeSO_4_ and glutamate, as described in Bogdan et al. (36), (iv) foaming was controlled by automatic addition of simethicone (30% emulsion [Dow Corning Q7-2587] diluted 2-fold in water), which is essentially similar in composition to Antifoam C (Sigma) used by Bogdan et al. (36), (v) the temperature was 36.5°C, (vi) a constant aeration rate of 4.0 liters · min^−1^ was used, and (vii) the level of dissolved oxygen was regulated at 40%.

## Supplementary Material

Supplemental material
