# Supplementary material for: Probing the Genome-Scale Metabolic Landscape of Bordetella pertussis, the Causative Agent of Whooping Cough
Source: Appl Environ Microbiol. 2017 Oct 17;83(21):e01528-17. doi: 10.1128/AEM.01528-17 (PMC5648915; doi:10.1128/AEM.01528-17)
Supplement: Supplemental material [file supp_83_21_e01528-17__index.html]

Supplemental material 

# Probing the Genome-Scale Metabolic Landscape of Bordetella pertussis, the Causative Agent of Whooping Cough

## Supplemental material

- Supplemental file 1 -

  Supplemental text; main characteristics of model iBP1870 (Table S1); net consumption/production of biomass and major extracellular metabolites in reference fermentations A and B (Fig. S1); schematic view of *B. pertussis* Tohama I biomass equation (Fig. S2); arginine and nucleobase production (Fig. S3); pseudocode representation of EMAF minimal medium-generating algorithm (Fig. S4); overview of minimal requirements of *B. pertussis* and other classical bordetellae for S, C/N, and C sources (Fig. S5).

  PDF, 1.9M
- Supplemental file 2 -

  Experimental procedures and resulting metabolite quantification data for reference fermentations A and B, as used for model construction and determination of ATP parameters (Data Set S1).

  XLSX, 24K
- Supplemental file 3 -

  Experimental procedures and biomass characterization data for reference fermentations A and B, as used for defining the biomass equation of *B. pertussis* Tohama I (Data Set S2).

  XLSX, 1.4M
- Supplemental file 4 -

  Detailed description of the metabolic network iBP1870, including literature information, gene content evidence, and experimental data used for network curation (Data Set S3).

  XLSX, 767K
- Supplemental file 5 -

  Data used for determination of ATP parameters for model iBP1870, based on the measured metabolic fluxes in reference fermentations A and B (Data Set S4).

  XLSX, 45K
- Supplemental file 6 -

  Data for the comparison of simulated and experimentally determined growth yields, using growth yield information from previously published work and from newly generated growth data (Data Set S5).

  XLSX, 30K
- Supplemental file 7 -

  Data from LC-MS based identification of N-containing end products (Data Set S6).

  XLSX, 741K
- Supplemental file 8 -

  Data from the simulations of minimal growth requirements of *B. pertussis* Tohama I using EMAF, including constraints and results (Data Set S7).

  XLSX, 1.3M
- Supplemental file 9 -

  Data from the reconstruction of the metabolic networks of 14 additional strains of *Bordetella* species, including the method used (Data Set S8).

  XLSX, 147K
