# Supplementary material for: Probing the Genome-Scale Metabolic Landscape of Bordetella pertussis, the Causative Agent of Whooping Cough
Source: Appl Environ Microbiol. 2017 Oct 17;83(21):e01528-17. doi: 10.1128/AEM.01528-17 (PMC5648915; doi:10.1128/AEM.01528-17)
Supplement: Supplemental material [file AEM.01528-17_zam999118131s1.pdf]

I. METABOLIC NETWORK CONSTRUCTION

The metabolic model of *B. pertussis* Tohama I was constructed in three phases: (i) draft semi-automatic model reconstruction, (ii) addition of newly defined pathways and reactions, and (iii) final manual curation.

I.1. Draft reconstruction of B. pertussis Tohama I metabolic network

A draft metabolic network of *B. pertussis* Tohama I was constructed by implementing the AUTOGRAPH method [1], using model iAF1260 for *E. coli* MG1655 as a template [2]. The reasons behind this choice have to do with the phylogenetic proximity between the two organisms (both are proteobacteria), and the general high quality of iAF1260, in which the criteria underlying the manual curation is easily traceable.

Pairwise orthologous relationships between the 3,436 open reading frames (ORF) of *B. pertussis* Tohama I (query species) and the 4,144 ORFs of *E. coli* MG1655 were established by comparing their genome sequences (retrieved in August 2010 from GenBank), resorting to the stand-alone version of Inparanoid (version 3.0) using BLOSUM45 as the substitution matrix [3]. Genes were considered orthologs if associated confidence percentage was higher than 95%. Extended orthologous relationships were established for paralogs, when a gene found to be ortholog to a gene in the other genome was also found to be a paralog again with a confidence percentage higher than 95%. This resulted in the creation of additional links for the *B. pertussis* loci BP3332 and BP1628, and for the *E. coli* loci b3787 and b0267. After sorting of inparanoid output based on the above criteria, we considered 1515 orthologous relationships between *B. pertussis* and *E. coli*. The original gene-reaction association of the genes found to be orthologous between the genomes of both strains was then transferred to the corresponding genes of the query species. This yielded the first fully-automated version of the metabolic network of *B. pertussis* Tohama I, which included 1665 tentative gene-reaction associations: 1,202 directly transferred based on orthology; and 463 originating from the inclusion of all non-gene associated reactions of iAF1260.

The curation of the first draft version of the model began by systematically comparing the original annotation of the genes as present in the GenBank files of *B. pertussis* and *E. coli*. This was found to be strictly identical for 180 of the 1,665 original associations. The remaining 1,485 associations of the fully automated model were further curated by manual inspection of the list of gene-reaction associations. In short, we ensured that the associated reaction equation was consistent with the genome annotation of *B. pertussis* and, when available, with the reaction information retrieved from BRENDA via the E.C. annotation in iAF1260. This was the case for an additional 949 entries. Out of the 536 reactions left, 463 were non-gene associated. The remaining 73 gene reaction associations involved genes annotated as hypothetical in 22 cases and were tentatively included in the model, but with both their upper and lower bounds closed.

At this stage, the curated list of reactions was made to be machine-readable following SBML3 standards using algorithms made available in the Open Source software, PySCeS CBMPy (version 0.7.0) [4]. Consistency between model simulations and experimental data was being considered in an iterative manner as previously described [5]. Details about model curation are presented in subsequent sections together with the supporting data.

I.2. Newly defined pathways and reactions

The draft metabolic network of *B. pertussis* Tohama I, as reconstructed based on the metabolic network of *E. coli*, lacks several essential pathways of *B. pertussis*, and is therefore incapable of (i) producing several key biomass constituents (*B. pertussis* specific lipooligosaccharide constituents, *B. pertussis* specific siderophores, biosynthesis of storage compounds such as PHB or polyphosphate) and (ii) accurately explaining the catabolism of several amino acids (such as branched-chain or aromatic amino acids) which are taken up in sometimes large excess compared to the biomass needs. The necessary pathways were reconstructed based on literature information and gene content, as described in details in Dataset S3.

I.3. Final manual curation

A final round of manual curation was performed with the updated model.

<u>I.3.1. “Orphan” metabolites and elemental/charge balances</u>

Before curation, 362 metabolites (out of 1646) were identified as “orphan” metabolites – i.e. metabolites that are the product or substrate of only one reaction in the network –, reflecting missing reactions in the network or “carry-over” reactions from the *E. coli* network. These metabolites were screened manually and decision was taken either to remove them from the network (together with the corresponding reactions) or to add the missing reaction(s). This was performed iteratively, on a case-by-case basis. In parallel, all reactions were checked for charge and atom balance, and corrected if required. Overall, this resulted in the deletion of 390 reactions and 396 metabolites, the

addition of 154 new reactions and 13 metabolites, and the modification of 30 reactions (name and/or equation) and 15 metabolites (name only).

Every reaction in the resulting network is elementary and charge balanced, and the network only contains 15 "orphan" metabolites, which were retained because they are the product/substrate of a gene-associated reaction (hence, there is a high confidence level for the presence of the reaction). These metabolites reflect different situations: (i) possible remaining gaps in the network which could not be filled based on genome sequence analysis alone (for instance, several acyl-coenzyme A species), (ii) absence of an external sink for the metabolite (for instance, biotin or several metals, which are not part of the biomass equation), or (iii) truly incomplete pathways such as NAD biosynthesis (hence, the auxotrophy of *B. pertussis* for niacin). For model-based identification of metabolic engineering strategies, for instance, it is important to keep such metabolites in the network, although the corresponding reactions would never carry a flux in the current version of the model. Also, these 15 metabolites represent good starting points for future improvement of the *B. pertussis* metabolic network.

I.3.2. Default constraints to account for physiological observations

Two features of the reconstructed network of *B. pertussis* Tohama I are in conflict with published physiological observations: (i) presence of a complete Krebs cycle and (ii) presence of a complete pathway for the biosynthesis of L-Cys from sulfate.

I.3.2.1. Tricarboxylic acid (TCA) cycle

Genes encoding all enzymes of the TCA cycle are present in the genome sequence (and therefore in the modeled metabolic network), whereas this cycle has been reported as partially dysfunctional, based on the observation that acetyl-coenzyme A and oxaloacetate cannot be converted to α-ketoglutarate [6]. Reactions corresponding to the dysfunctional part of the TCA cycle are R_CS (M_accoa + M_oaa + M_h2o => M_cit + M_coa + M_h), R_ACONTa (M_cit <=> M_acon_C + M_h2o), R_ACONTb (M_acon_C + M_h2o <=> M_icit), R_ICDHyr (M_icit + M_nadp <=> M_akg + M_co2 + M_nadph). In order to account for the physiological observation of Thalen *et al.* [6], these 4 reactions were constrained in order not to function in the direction from oxaloacetate to α-ketoglutarate (i.e. R_CS: 0,0; R_ACONTa, R_ACONTb, and R_ICDHyr: -99999,0). Flux balance analysis (FBA) was then performed on different model versions, using the external flux constraints derived from measurements in the reference fermentations (Dataset S1), in order to evaluate the impact on (i) growth feasibility and (ii) the energetics of the cell (i.e. how much ATP can be produced). No impact on the feasibility of growth was found with a partially dysfunctional Krebs cycle. However, a significant impact was observed on the maximum possible energy production, depending on the constraints imposed on the Krebs cycle. Constraining either R_CS, R_ACONTa, or R_ACONTb (i.e. any of the reactions that leads from oxaloacetate to isocitrate) results in an equivalent impact on ATP production (19% decrease), while the impact of constraining only R_ICDHyr is less dramatic (10% decrease).

In view of the potentially big impact on energy parameters, the abovementioned constraints for all 4 reactions were implemented by default in the model, i.e. the default model was constrained to have a partially dysfunctional Krebs cycle.

I.3.2.2. L-Cys biosynthesis from inorganic sulfate

Because the full sequence of reactions for the L-Cys biosynthesis pathway from sulfate via 3'-phosphoadenylyl sulfate, is present in the reconstructed *B. pertussis* Tohama I metabolic network, growth with sulfate as the sole source of S would be feasible *in silico*. However, this would be in contradiction with many reports about the growth requirements of *B. pertussis* [7,8]. Based on these physiological observations and on the fact that two of the genes encoding reactions in the pathway from sulfate to L-Cys are pseudogenes (BP0970, encoding CysH, and BP0971, encoding CysN [8]), default constraints were set on the corresponding reactions (R_PAPSR, R_PAPSR2, and R_ADSK) so that no flux is possible through this pathway. This was verified with FBA: no biomass production was possible with sulfate as the sole S source when these 3 reactions were constrained.

II. DETERMINATION OF ENERGY PARAMETERS

II.1. Definitions

Metabolic energy is classically divided between the demands of biosynthetic processes (growth) and those of non-growth associated processes [9], which are traditionally represented by the growth yield (Y^{ATP}; g_{DCW} $mmol_{ATP}^{-1}$) and the maintenance coefficient (m_{ATP}; $mmol_{ATP}$ g_{DCW}^{-1} h^{-1}), respectively. This can be represented by the following relation:

$$q_{ATP} = m_{ATP} + \frac{\mu}{Y^{ATP}} \quad (1)$$

where q_{ATP} represents the specific ATP consumption rate ($mmol_{ATP}$ g_{DCW}^{-1} h^{-1}) and μ is the specific growth rate (h^{-1}).

A fraction of the growth-associated ATP consumption (termed x; $mmol_{ATP}$ g_{DCW}^{-1}) is expcitly included in the *B. pertussis* reaction network in the reactions leading to the synthesis of biomass precursors. However, growth (i.e. the net production of new biomass) also requires additional ATP expenses, which are not explicitly taken into account in the model. This non-explicit ATP consumption (termed y; $mmol_{ATP}$ g_{DCW}^{-1}) covers the amount of ATP necessary for the assembly of biomass precursors into new cells [10]. The growth yield Y^{ATP}, the total growth-associated ATP consumption (GAM_{TOT}; $mmol_{ATP}$ g_{DCW}^{-1}), and parameters x and y are related as follows:

$$GAM_{TOT} = x + y = \frac{1}{Y^{ATP}} \quad (2)$$

From a modeling point of view, x, the explicit growth-associated ATP consumption, is intrinsically taken into account in the model. The non-explicit growth-associated ATP consumption, y, can be introduced as an ATP hydrolysis reaction within the biomass equation and has units $mmol_{ATP}$ g_{DCW}^{-1} [10]. The maintenance ATP consumption (i.e. non-growth associated) can be modeled as an independent ATP hydrolysis reaction (R_ATPM: M_atp_c + M_h2o_c => M_adp_c + M_pi_c + M_h_c), the flux through which represents maintenance [10]. Units of the flux through the maintenance reaction should be identical to units of the other fluxes.

Conversion of specific rates into concentrations (or absolute amounts) in equation 1 results in the following equation:

$$ATP_{tot} = (X_{t=0}e^{\mu t} - X_{t=0})\left(\frac{m_{ATP}}{\mu} + \frac{1}{Y^{ATP}}\right) \quad (3)$$

where ATP_{tot} represents the total ATP consumption ($mmol_{ATP}$ L^{-1} or $mmol_{ATP}$), $X_{t=0}$ is the biomass concentration (or amount) at the start of fermentation (g_{DCW} L^{-1} or g_{DCW}), and t is the cultivation time (h). Importantly, equation 3 implies a constant specific growth rate, i.e. exponential growth.

Equation 3 can also be written in terms of x and y (see equation 2):

$$ATP_{tot} = (X_{t=0}e^{\mu t} - X_{t=0})\left(\frac{m_{ATP}}{\mu} + x + y\right) \quad (4)$$

II.2. Determination of m_{ATP} and Y^{ATP}

A widely used method for determining the value of m_{ATP} and Y^{ATP} is from the specific ATP consumption rates at different dilution rates (specific growth rates) in chemostat cultures. Assuming a linear relationship between ATP consumption rate and growth rate (see equation 1), the growth yield (Y^{ATP}; g_{DCW} $mmol_{ATP}^{-1}$) corresponds to $slope^{-1}$ of this relationship, and the maintenance energy (m_{ATP}; $mmol_{ATP}$ g_{DCW}^{-1} h^{-1}) to the intercept with the y-axis (ATP consumption at zero growth). ATP consumption can be calculated considered equal to ATP that production under the assumption of full coupling between catabolic ATP supply and anabolic ATP demand.

In the present study, the two reference fermentations are batch fermentations, and no chemostat data were generated. The total amount of ATP generated (ATP_{tot}; $mmol_{ATP}$) was calculated independently *for each phase* by using Flux Balance Analysis (FBA) to maximize the flux through reaction R_ATPM (an ATP hydrolysis reaction), using a model version without a functional Krebs cycle, with a biomass equation containing no growth-associated ATP hydrolysis (y=0), and with constraints reflecting the measured substrates and products (full description of constraints available in Dataset S4).

The maximum possible ATP production for every phase of the two fermentations, as calculated from FBA, is summarized in Dataset S4. These values represent y, the fraction of ATP consumption that is not explicitly taken into account in the model (i.e. maintenance and assembly of the biomass precursors into cells). From the same FBA simulations, the fraction of growth-associated ATP consumption which is explicitly taken into account in the model in the form of biomass precursor biosynthesis (x), was calculated as the reduced cost associated to the biomass equation ($mmol_{ATP}$ g_{DCW}^{-1}), multiplied by the biomass produced in every phase (g_{DCW}).

In the next step, the growth yield (Y^{ATP}; g_{DCW} $mmol_{ATP}^{-1}$) and maintenance ATP requirements (m_{ATP}; $mmol_{ATP}$ g_{DCW}^{-1} h^{-1}) were estimated from the calculated total ATP consumption and from the measured biomass production of individual phases. This was done by fitting values for parameters Y^{ATP} and m_{ATP}, so as to minimize the sum of squared differences

between FBA-derived total ATP consumption and calculated total ATP consumption (using equation 3), with the Solver function in Excel. Importantly, the use of equation 3 implies that growth during each metabolic phase follows an exponential growth pattern at a constant μ. The specific growth rate was calculated individually for every phase, based on the corresponding initial and final biomass amounts (Dataset S4).

The deduced values are m_{ATP} = 9.21 $mmol_{ATP}$ g_{DCW}^{-1} h^{-1} and Y^{ATP} = 0.0036 g_{DCW} $mmol_{ATP}^{-1}$.

II.3. Comparison of energy parameters with published values

In the literature, only one report is available for the determination of maintenance and yield parameter values of *B. pertussis* using glutamate-limited chemostat data at different dilution rates [11]. Other *B. pertussis* reports such as those of Thalen *et al.* [6] or Frohlich *et al.* [12] only provide global substrate requirements in the form of an *apparent* growth yield, i.e. a lumped factor including growth-associated and maintenance requirements.

The energy parameters of Licari *et al.* [11] are expressed as a function of substrate amounts (i.e. m_s and Y^{xs}) rather than ATP amounts. Conversion of these parameters is therefore needed, which first requires computing the maximum yield of ATP per L-Glu. This was calculated using the metabolic model, by maximizing the amount of ATP produced (using Flux Balance Analysis) with L-Glu as the sole substrate, unlimited oxygen supply, and no constraint on the metabolic end-products: a value of 15.5 mol_{ATP} per $mol_{L\text{-}Glu}$ was determined for the yield of ATP on L-Glu. The resulting energy parameters are m_{ATP} = 0.83 $mmol_{ATP}$ g_{DCW}^{-1} h^{-1} and Y^{ATP} = 0.0027 g_{DCW} $mmol_{ATP}^{-1}$ (Dataset S4).

II.4. Implementation of energy parameters into the model

For implementing energy parameters into the metabolic model, parameter Y^{ATP} cannot be used as such, but must be divided into explicit (*x*) and non-explicit (*y*) growth-associated ATP demand (equation 2). The value of *y* was obtained using equation 2, from the calculated value for Y^{ATP} (0.0036 g_{DCW} $mmol_{ATP}^{-1}$) and the explicit ATP consumption obtained from FBA (*x*=99.75 $mmol_{ATP}$ g_{DCW}^{-1}; average of the overall ATP consumption in the two reference fermentations; Dataset S4). The non-explicit ATP demand *y* (179.27 $mmol_{ATP}$ g_{DCW}^{-1}; Dataset S4) was introduced as the stoichiometric coefficient for ATP hydrolysis in the biomass equation (see model iBP1870):

5.5004472994 M_prot_BP_c + 0.2097650049 M_DNA_BP_c + 0.1258750321 M_RNA_BP_c + 1.0 M_LIPID_BP_c + 1.0 M_PG_BP_p + 1.0 M_LPS_BP_e + 1.0 M_COFACTORS_BP_c + 1.0 M_IONS_BP_c + 0.0011594203 M_phb_c + ***179.2677995561 M_atp_c + 179.2677995561 M_h2o_c*** *=>* ***179.2677995561 M_adp_c + 179.2677995561 M_pi_c + 179.2677995561 M_h_c*** *+ 1.0 M_BIOMASS_c*

When working with specific rates as constraints, the calculated value for the maintenance coefficient m_{ATP} (9.21 $mmol_{ATP}$ g_{DCW}^{-1} h^{-1}) can be introduced as such as a lower bound to the R_ATPM ATP hydrolysis reaction (M_atp_c + M_h2o_c => M_adp_c + M_pi_c + M_h_c).

However, when working with concentrations or absolute amounts as flux constraints – as in the case of calculating the biomass production of batch cultures –, the total amount of ATP used for maintenance purposes must be calculated. This can be done with the following equaton (maintenance part of equation 3):

$$ATP_{maintenance} = (X_{t=0}e^{\mu t} - X_{t=0})\left(\frac{m_{ATP}}{\mu}\right) \qquad (5)$$

For the reference fermentations, $ATP_{maintenance}$ was calculated based on the measured values for $X_{t=0}$, *t* and μ, and equals 10235.30 $mmol_{ATP}$ or 890.03 $mmol_{ATP}$ L^{-1} (average of the two reference fermentations; Dataset S4). The calculated value for $ATP_{maintenance}$ can be introduced as a lower bound constraint to the R_ATPM reaction.

III. PREDICTION OF MINIMAL GROWTH REQUIREMENTS

Specifically, we pose the question: is it possible to list the minimal growth requirements that will enable a specific yield? In this context, we define growth as biomass yield per unit substrate uptake and minimal growth requirements as the simplest set of substrates necessary to achieve a target biomass yield.

III.1. Modeling framework and software

In the discussion that follows, a medium component is represented by an uptake flux, i.e. if substrate X is required for growth in the predicted medium, this is modeled as a required non-zero flux through the exchange reaction for X.

All modeling and algorithms described here were developed and implemented using the Open Source software, PySCeS CBMPy (version 0.7.0) making use of the IBM ILOG CPLEX™ 12.5.1 (Academic Edition) for optimization [4]. CBMPy is a Python-based modeling environment and examples of the scripts used to generate the results shown below are available upon request.

III.2. Algorithm for the calculation of the minimal number of substrates

A set of import reactions that define the minimal growth requirements can be used to conceive a minimal growth medium, i.e. one that contains the fewest number of added components. We adopt the hypothesis that, with respect to transport reactions, it is more likely that the cell minimizes the number of active transporters used to achieve a specific objective, rather than the combined flux passing through them. A minimal medium is therefore defined as a medium which supports the minimum number of active transporters (uptake) needed to satisfy a particular objective, in this case biomass production.

In order to compute such a minimal medium, we make use of a mixed integer linear program (MILP) based on the standard FBA LP formulation (constraints and flux bounds) but in addition introduce binary variables to control whether a flux is on (a non-zero flux) or off, and a constraint that fixes the objective to a specific target value. This MILP is described in equation B and is analogous to a previously used approach [13].

$$
\begin{aligned}
&\text{minimize: } \sum_{1}^{i} z_i \\
&\text{such that:} \\
&NJ = 0 \\
&J_{opt} \geq optarg \\
&z_i = 0 \rightarrow J_i = 0 \\
&\\
&lb_n \leq J_n \leq ub_n \\
&z_i \in \{0 : 1\}
\end{aligned}
$$

Equation B. MILP minimizing the number of non-zero fluxes. J_n represents the variable model fluxes, *N* the stoichiometric matrix, J_{opt} is the FBA objective function flux, *optarg* is the target objective function value, *lb* and *ub* are constants representing the lower and upper bounds of each flux and *z* is a binary indicator variable which is zero if the constraint it represents is active. The objective function minimizes the number of non-zero indicator variables i.e. non-zero fluxes, in this case the set of fluxes represented by J_i.

There are various ways of implementing the MILP shown in equation B, the conventional approach being to use a so-called Big M formulation. However, Big M methods can be sensitive to the values of the artificial parameters used to turn on or off a constraint, and therefore, may lead to problems involving numerical instability (IBM ILOG CPLEX™ Optimization Studio 12.5.1. 2013. http://www-03.ibm.com/software/products/en/ibmilogcpleoptistud/). By developing a custom implementation using the IBM ILOG CPLEX™ optimizer and formulating the problem using *indicator constraints* it was possible to avoid an explicit Big M formulation and thus exploit CPLEX's ability to minimize these side-effects (IBM ILOG CPLEX™ Optimization Studio 12.5.1. 2013. Reference Manual, Indicator constraints in optimization http://www-03.ibm.com/software/products/en/ibmilogcpleoptistud/). This implementation proved to be both fast and efficient in calculating a set of minimal growth requirements and showed that it was computationally feasible to compute a minimal medium. The algorithm provides a single solution (medium composition) to the problem, from which the number of active fluxes can be deduced.

III.3. Algorithm for the enumeration of minimal active fluxes

While the algorithm described above can provide a single solution (i.e. a single medium composition) that satisfy the condition of the minimal number of active fluxes, there may be alternative solutions (media compositions) that satisfy

this same optimal criterion. In this paragraph, we describe the development of an algorithm to enumerate all possible substrate combinations that have the same, minimal number of active uptake fluxes.

In general, the enumeration of MILP solution spaces is a computationally hard problem. Here, we developed a strategy that takes advantage of the initial solution provided by the MILP optimization: by limiting the scope of our search to only find combinations with a set (minimal) number of active transport reactions, the search space is reduced so as to become computationally feasible.

The EMAF algorithm (for Enumeration of Minimal Active Fluxes) is essentially composed of three parts, each represented as pseudo-code in Fig. S4 and examples of which are available upon request.

To begin with, EMAF initializes the model, sets up the external environment and performs both an active flux minimization and flux variability analysis (FVA) on the target reactions (in this case, the import reactions). This provides the global minimum number of reaction (medium components) as well as a set of *required reactions* defined as: reactions that have an FVA minimum greater than zero or in other words reactions that are required to carry a flux in all solutions (auxotrophies). It then calls the *Subsearch* function recursively.

The *Subsearch* function is the core of the algorithm and takes as arguments a model instance, minimization target and a list of reactions to ignore. Essentially *Subsearch* computes minimal number of active flux solutions and will exit if the model is either infeasible or the number of active solutions is larger than the global minimum. It then finds the set of non-zero fluxes, as specified by the target list and not in the ignore list (*Subsearch* will return if target set is empty) and labels them as testable.

For each flux in testable, clone the model, set its bounds to zero and all other fluxes in testable to a small positive number, add them to a new ignore list and call *Subsearch* recursively using the new model, target and ignore parameters. When *Subsearch* fails any of the tests mentioned earlier, it returns either an empty solution or the testable flux it was evaluating (set to zero) which results in a depth-first search of the minimal media solution space.

The final part of the algorithm, *ConstructMedia* unrolls the recursive data structures and regenerates the qualitative media compositions. It then uses this information coupled with an absolute flux minimization approach to quantify the minimal flux required to be taken up to achieve the target objective. For this particular model, while not formally proven, EMAF appears to enumerate all minimal media required to satisfy a target objective.

III.4. Prediction of minimal growth requirements for Tohama I

EMAF was used to identify all possible sets of nutrients – among pre-defined possible nutrients – required for producing *B. pertussis* Tohama I biomass, with the least possible number of active input fluxes (i.e. least possible number of nutrients). Specifically, in the present study, we applied EMAF to identify possible S sources, possible amino acids as C/N sources, and possible organic acids as C sources.

Practically, for each simulation, EMAF was provided with the following informations: (i) the *metabolic model* of *B. pertussis* Tohama I, (ii) a *constraint file of input fluxes*, reflecting the possible nutrients to choose from (including the maximal concentration allowed; Dataset S7), (iii) the *list of target reactions* to be minimized (i.e. the list of reactions from which the minimal number of active fluxes must be identified; in this case, all import reactions (R_EXi_) except the import reactions for , O_2, and H^+; Dataset S7), and (iv) the objective function to be maximized (in this case, the biomass reaction *R_BP_biomass_130704*, subject to the following constraints: LB=UB=1).

For this application, we used a version of model iBP1870 where exchange reactions – usually modeled as reversible (“R_EX”) – have been split into individual irreversible import (“R_EXi_”) and export (“R_EXo”) reactions (“split” model version, available from https://github.com/SystemsBioinformatics/pub-data/tree/master/bordetella-pertussis-model). This allows to specifically minimize the number of *input* fluxes (i.e. nutrients), with no constraint on the number of *output* fluxes (i.e. metabolic end-products). All simulations were performed in duplicate, using a model with or without additional constraints on the R_CS, R_ACONTa, R_ACONTb and R_ICDHyr reactions to simulate the impact of a partially non-functional TCA cycle R_CS: 0,0; R_ACONTa, R_ACONTb, and R_ICDHyr: -99999,0) vs. a fully functional TCA cycle (R_CS: 0,99999; R_ACONTa, R_ACONTb, and R_ICDHyr: -99999,99999).

As an output of EMAF, the flux distribution for each possible combination of nutrients resulting in the least number of input fluxes, was obtained (Dataset S7).

IV. SUPPLEMENTAL TABLES

Table S1. Main characteristics of model iBP1870

	**"unsplit" model[a]**	**"split" model[a]**
Included genes	762 (22%)	762 (22%)
Reactions	1,675	1,877
Exchange reactions	202	404
Demand reactions	5	5
Macromolecule synthesis reactions[b]	9	9
Internal reactions	1,459	1,459
Transport reactions	583	583
Metabolic reactions	876	876
Gene-associated reactions	1,017 (70%)	1,017 (70%)
Metabolites		
Unique metabolites	818	818
Cytoplasm	759	759
Periplasm	286	286
Extracellular	209	209

[a]the "unsplit" and "split" models only differ in the number and definition of exchange reactions. In the "unsplit" model, exchange reactions are defined as reversible reactions (termed R_EX_), and can account for both the input and output of metabolites into the system. In the "split" model, exchange reactions are irreversible, and each R_EX_ reaction has been split into a reaction that accounts for the input of the metabolite into the model (R_EXi_) and a reaction that accounts for the output of the metabolite (R_EXo_).

[b]macromolecule synthesis reactions are defined as the biomass equation itself as well as reactions for the synthesis of macromolecules (DNA, RNA, proteins, peptidoglycan, lipids, lipopolysaccharide, inorganic ions pool, and cofactors pool) that are part of the biomass.

V. SUPPLEMENTAL FIGURES

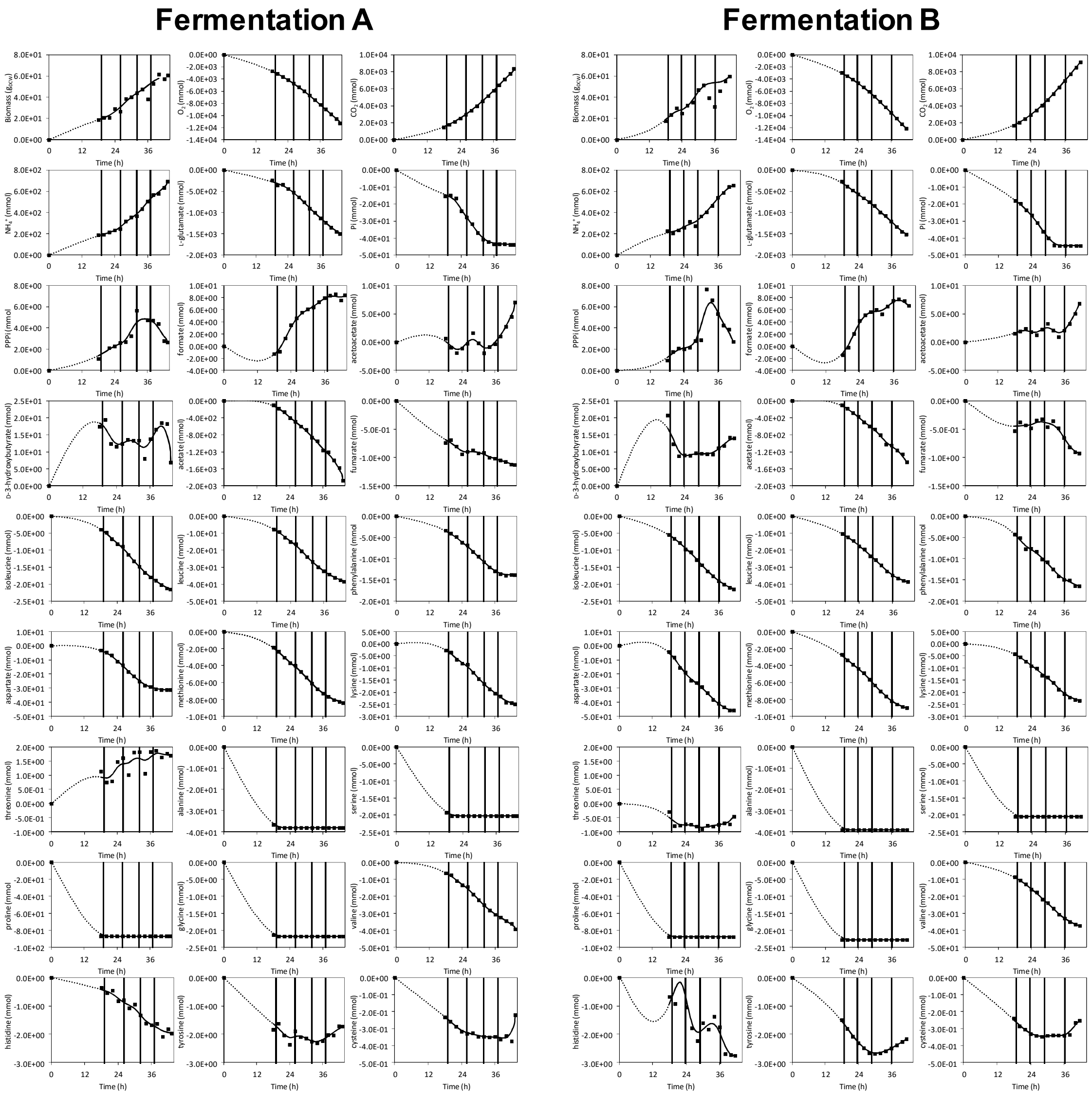

Figure S1. Net consumption/production of biomass and major extracellular metabolites in reference fermentations A and B (used for model construction and calibration).

The net consumption/production (expressed in mmol) represents the total amount consumed/produced from the start of the fermentation, in the entire culture volume, at each time-point. Symbols represent the measured net consumption/production of metabolites (available in Dataset S1), vertical lines represent metabolic phase boundaries, solid lines represent fitted spline functions, and dotted lines represent the spline function for the initial phase (Phase 0), during which no sampling was performed. Ten additional metabolites were measured but their concentration was found to be very low. Polyphosphate (PPPi) is intracellular: because the polyphosphate content of biomass fluctuates with time, it was not included in the biomass equation; rather, the amount of intracellular polyphosphate was converted to extracellular equivalents of a triphosphate molecule.

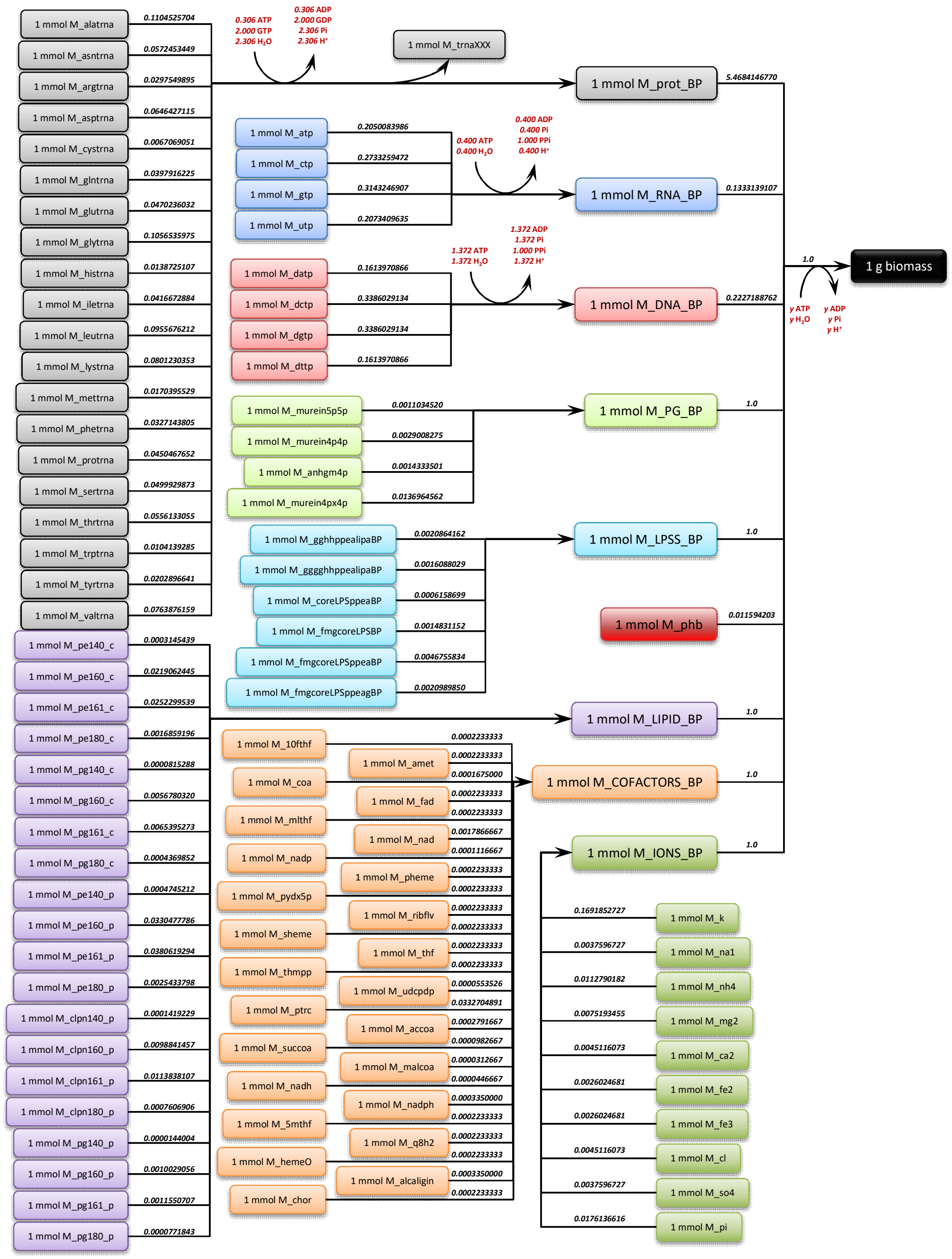

Figure S2. Schematic view of *B. pertussis* Tohama I biomass equation.

Reactions in the model are represented by lines, together with the stoichiometric coefficients. Metabolites, macromolecules, and pools of metabolites are represented by squares, whose color reflects the macromolecule or pool of metabolites (grey, proteins; blue, RNA; light red, DNA; light green, peptidoglycan; light blue, lipopolysaccharide; red, poly-β-hydroxybutyrate; purple, lipids; orange, cofactors; green, inorganic ions). Metabolite nomenclature is as in the model (*M_aaa[_BP]_b*, where *aaa* is the metabolite abbreviation and *b* indicates the cellular compartment (omitted for all groups except lipids)). For protein, RNA, and DNA synthesis reactions, as well as for biomass assembly, hydrolysis of ATP/GTP, and release of inorganic pyrophosphate (PPi), is indicated in red, with the associated stoichiometric coefficients. In the case of biomass synthesis, the stoichiometric coefficient *y* represents the growth-associated ATP consumption. Determination of the value of *y* is described in supplemental text and Dataset S4. For protein synthesis, the release of uncharged tRNA (*M_trnaxxx*, where *xxx* is an amino acid) is not explicitly depicted for every amino acid.

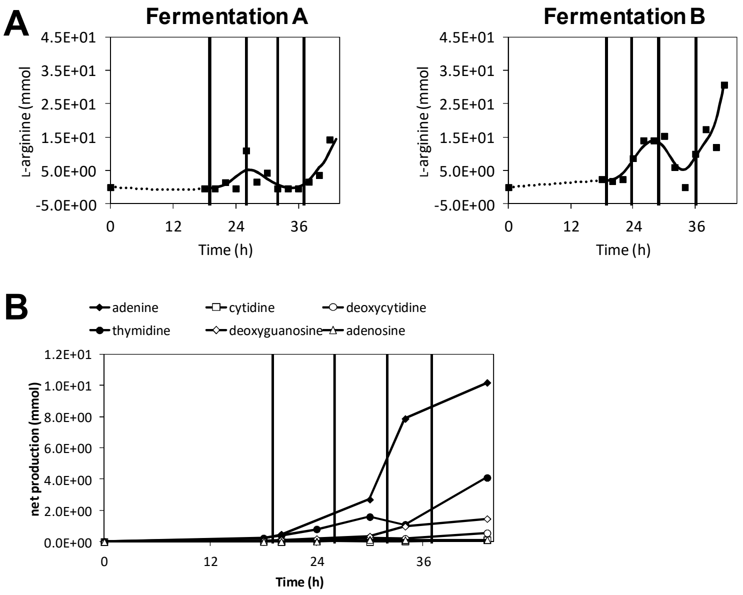

Figure S3. Arginine and nucleobase production by *B. pertussis*.

A. Net production of L-arginine in reference fermentations A and B. Symbols represent the measured net consumption/production of metabolites, vertical lines represent metabolic phase boundaries, solid lines represent fitted spline functions, and dotted lines represent the spline function for the initial phase (Phase 0), during which no sampling was performed. **B.** Net production of nucleobases in reference fermentation A (LC-MS based quantification).

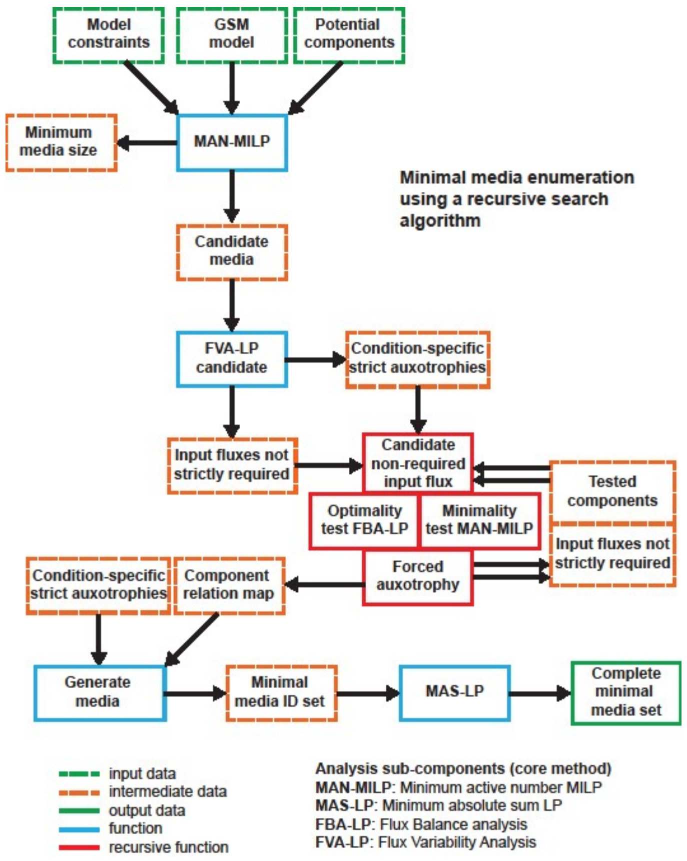

Figure S4. Pseudo-code representation of EMAF minimal media generating algorithm.

A targeted, recursive media search algorithm is implemented in three parts. An EMAF driver method that initializes the model, determines the initial search parameters and initiates a recursive *Subsearch* which through a process of elimination determines minimal viable media combinations. Finally in *ConstructMedia,* minimal media combinations are unrolled and quantified to construct minimal media sets. This algorithm has been fully implemented using CBMPy.

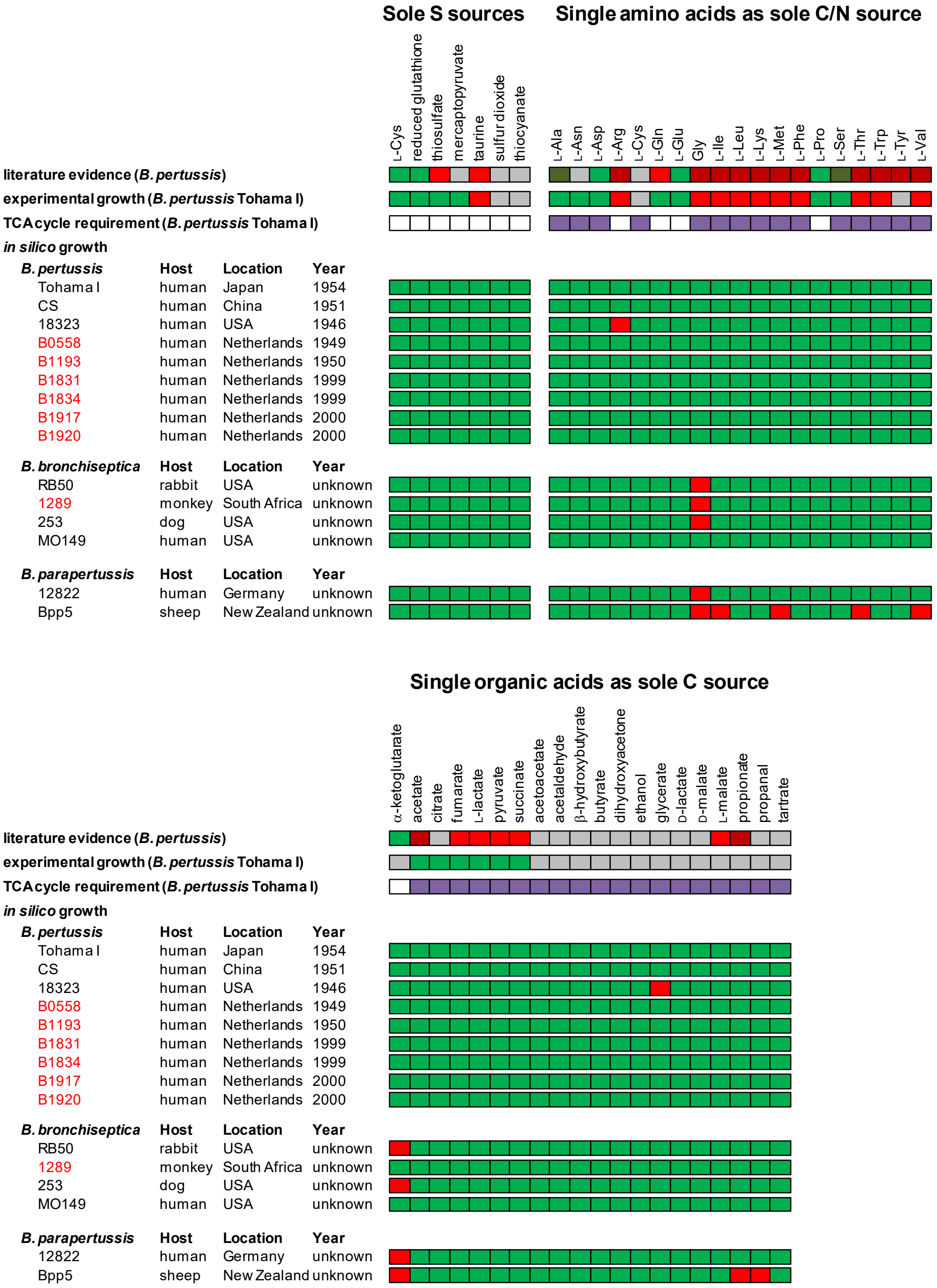

Figure S5. Overview of minimal requirements of *B. pertussis* and other classical bordetellae for S, C/N, and C sources.

Strain names in red refer to strains for which the available genome sequence is made of more than one contig/scaffold. Color code: green, growth; red, no growth; gray, no data available (literature evidence) or not tested (experimental growth); dark red and dark green (literature evidence only), literature data are not conclusive in terms of minimal growth requirements since the only available data for catabolism of these amino acids or organic acids is from experiments with cell suspensions: no literature data available about growth of *B. pertussis* with these substrates as sole C or C/N source (see supplemental text). Color code for TCA cycle requirements: empty, no absolute requirement for a functional TCA cycle; purple, absolute requirement for a fully functional TCA cycle, i.e. no *in silico* growth possible in the absence of reactions R_CS, R_ACONTa, R_ACONTb, and R_ICDHyr. Only the experimentally verified nutrient requirements of Tohama I are indicated (for experimental verification with other strains, refer to Fig. 5D).

VI. DESCRIPTION OF OTHER SUPPLEMENTAL FILES

Dataset S1. Data from reference fermentations A and B (Microsoft Excel workbook - .xlsx). Includes the experimental procedures and resulting metabolite quantification data for reference fermentations A and B, as used for model construction and determination of ATP parameters.

Dataset S2. Biomass composition of *B. pertussis* (Microsoft Excel workbook - .xlsx). This file contains the experimental procedures and biomass characterization data for reference fermentations A and B, as used for defining the biomass equation of *B. pertussis* Tohama I.

Dataset S3. Detailed description of the reconstructed metabolic network of *B. pertussis* Tohama I (model iBP1870) (Microsoft Excel workbook - .xlsx). This file contains a detailed description of the metabolic network iBP1870, including literature information, gene content evidence, and experimental data used for network curation.

Dataset S4. Calculation of energy parameters (Microsoft Excel workbook - .xlsx). This file contains the data used for determination of ATP parameters for model iBP1870, based on the measured metabolic fluxes in reference fermentations A and B.

Dataset S5. Validation of model iBP1870 against published and newly generated datasets (Microsoft Excel workbook - .xlsx). This file contains data for the comparison of simulated and experimentally determined growth yields, using growth yield information from previously published work and from newly generated growth data.

Dataset S6. Identification of N sinks (Microsoft Excel workbook - .xslx). This file contains the data from LC-MS based identification of N-containing end-products.

Dataset S7. Prediction of minimal growth requirements of *B. pertussis* Tohama I using EMAF (Microsoft Excel workbook - .xlsx). This file contains the data from the simulations of minimal growth requirements of B. pertussis Tohama I using EMAF, including constraints and results.

Dataset S8. Metabolic reconstructions for 14 additional strains of classical bordetellae (Microsoft Excel workbook - .xlsx). This file contains data from the reconstruction of the metabolic networks of 14 additional strains of *Bordetella* sp., including the method used.

Model iBP1870 can be downloaded from https://github.com/SystemsBioinformatics/pub-data/tree/master/bordetella-pertussis-model (different formats available, including SBML). This version of the model contains default constraints reflecting a partially dysfunctional Krebs (TCA) cycle, the biomass equation with ATP consumption as defined in the supplemental material, and exchange reactions split between input and output.

VII. SUPPLEMENTAL REFERENCES

1. Notebaart RA, van Enckevort FH, Francke C, Siezen RJ, Teusink B (2006) Accelerating the reconstruction of genome-scale metabolic networks. BMC Bioinformatics 7: 296. 1471-2105-7-296

2. Feist AM, Henry CS, Reed JL, Krummenacker M, Joyce AR, Karp PD, Broadbelt LJ, Hatzimanikatis V, Palsson BO (2007) A genome-scale metabolic reconstruction for *Escherichia coli* K-12 MG1655 that accounts for 1260 ORFs and thermodynamic information. Mol Syst Biol 3: 121

3. Berglund AC, Sjolund E, Ostlund G, Sonnhammer EL (2008) InParanoid 6: eukaryotic ortholog clusters with inparalogs. Nucleic Acids Res 36: D263-D266

4. Olivier BG, Swat MJ, Mone MJ (2016) Modeling and Simulation Tools: From Systems Biology to Systems Medicine. Methods Mol Biol 1386: 441-463

5. Santos F, Boele J, Teusink B (2011) A practical guide to genome-scale metabolic models and their analysis. Methods Enzymol 500: 509-532

6. Thalen M, van den IJ, Jiskoot W, Zomer B, Roholl P, de GC, Beuvery C, Tramper J (1999) Rational medium design for *Bordetella pertussis*: basic metabolism. J Biotechnol 75: 147-159

7. Jebb WH, Tomlinson AH (1957) The minimal amino acid requirements of *Haemophilus pertussis*. J Gen Microbiol 17: 59-63

8. Parkhill J, Sebaihia M, Preston A, Murphy LD, Thomson N, Harris DE, Holden MT, Churcher CM, Bentley SD, Mungall KL, Cerdeno-Tarraga AM, Temple L, James K, Harris B, Quail MA, Achtman M, Atkin R, Baker S, Basham D, Bason N, Cherevach I, Chillingworth T, Collins M, Cronin A, Davis P, Doggett J, Feltwell T, Goble A, Hamlin N, Hauser H, Holroyd S, Jagels K, Leather S, Moule S, Norberczak H, O'Neil S, Ormond D, Price C, Rabbinowitsch E, Rutter S, Sanders M, Saunders D, Seeger K, Sharp S, Simmonds M, Skelton J, Squares R, Squares S, Stevens K, Unwin L, Whitehead S, Barrell BG, Maskell DJ (2003) Comparative analysis of the genome sequences of *Bordetella pertussis*, *Bordetella parapertussis* and *Bordetella bronchiseptica*. Nat Genet 35: 32-40

9. Pirt SJ (1965) The maintenance energy of bacteria in growing cultures. Proc R Soc Lond B Biol Sci 163: 224-231

10. Teusink B, Wiersma A, Molenaar D, Francke C, de Vos WM, Siezen RJ, Smid EJ (2006) Analysis of growth of *Lactobacillus plantarum* WCFS1 on a complex medium using a genome-scale metabolic model. J Biol Chem 281: 40041-40048

11. Licari P, Siber GR, Swartz R (1991) Production of cell mass and pertussis toxin by *Bordetella pertussis*. J Biotechnol 20: 117-129

12. Frohlich BT, De Bernardez Clark ER, Siber GR, Swartz RW (1995) Improved pertussis toxin production by *Bordetella pertussis* through adjusting the growth medium's ionic composition. J Biotechnol 39: 205-219

13. Klitgord N, Segre D (2010) Environments that induce synthetic microbial ecosystems. PLoS Comput Biol 6: e1001002
